# Noninvasive neurophysiological diagnostics of delayed cerebral ischemia in aneurysmal subarachnoid hemorrhage: a scoping review

**DOI:** 10.1007/s00701-026-06901-8

**Published:** 2026-05-11

**Authors:** J. Joep van der Harst, Johanna Hijlkema, Sjoukje van der Werf, Jan Willem J. Elting, J. Marc C. van Dijk, Maarten Uyttenboogaart

**Affiliations:** 1 Department of Neurology, University of Groningen, University Medical Center Groningen, PO Box 30.001, 9700 RB, AB51 Groningen, The Netherlands; 2 Medical Library, University of Groningen, University Medical Center Groningen, Groningen, The Netherlands; 3 Department of Neurosurgery, University of Groningen, University Medical Center Groningen, Groningen, The Netherlands

**Keywords:** Scoping review, Aneurysmal subarachnoid hemorrhage, Delayed cerebral ischemia, Transcranial doppler, Near infrared spectroscopy, Electroencephalography

## Abstract

**Background:**

Delayed cerebral ischemia (DCI) is a major complication following aneurysmal subarachnoid hemorrhage (aSAH), affecting outcomes. Given its multifactorial pathophysiology, including vasospasm, microcirculatory dysfunction, and neuroelectric disturbances, a range of diagnostic modalities has been studied. Neurophysiological techniques such as transcranial Doppler (TCD), electroencephalography (EEG), and near-infrared spectroscopy (NIRS) offer complementary insights. This scoping review provides an overview of these modalities, their analytical approaches, diagnostic performance, and temporal aspects of signal change in predicting DCI.

**Methods:**

The review followed PRISMA-ScR guidelines. A systematic search of PubMed, EMBASE, and Web of Science identified studies published between 2004 and January 2025. Eligible studies reported sensitivity, specificity, and/or area under the ROC curve (AUC) for predicting DCI or related outcomes in adult aSAH patients. 46 studies were included: 26 on TCD, 14 on EEG, and 6 on NIRS.

**Results:**

TCD was the most studied modality. Mean flow velocity (MFV) of the middle cerebral artery (MCA) was most frequently analyzed, with AUCs ranging from 0.59 to 0.81. Combining TCD with other variables, such as melatonin levels or qEEG features, improved diagnostic accuracy (AUC up to 0.96). NIRS studies used ROC-based rSO₂ cutoffs (65–70%) with AUCs up to 0.93. EEG studies using quantitative features, especially alpha/delta ratio, showed strong predictive value. Diagnostic performance should be interpreted in the context of heterogeneous outcome definitions across studies.

**Conclusion:**

TCD, EEG, and NIRS each contribute unique physiological data. Signal changes may precede DCI, suggesting a potential window for early intervention. Combined use may enhance early DCI detection. Future research should focus on multimodal integration, threshold standardization, and real-time predictive modeling, including artificial intelligence (AI) based approaches.

**Supplementary Information:**

The online version contains supplementary material available at 10.1007/s00701-026-06901-8.

## Introduction

Aneurysmal subarachnoid hemorrhage (aSAH) is a severe neurovascular condition. Advances in diagnostics and treatment have led to decrease in case fatality rates [33, 43]. Despite these advances, the burden of aSAH remains high [17, 43].

Clinical outcomes can be significantly improved through optimal timing of aneurysm repair to prevent rebleeding [24, 28, 75], as well as through proactive management of secondary complications such as elevated intracranial pressure, hydrocephalus, seizures, infections, and delayed cerebral ischemia (DCI). Current guidelines recommend a combination of preventive and surveillance strategies to mitigate the risk of DCI [24, 31].

After patients survive the acute phase and its associated complications, approximately one-third will develop DCI, particularly during the 4 to 14 days following ictus [16, 20, 31]. Notably, DCI-related infarction has been associated with an approximately five-fold higher likelihood of an unfavorable outcome at one year [80].

### Rationale

DCI represents a major secondary complication in aSAH, with a substantial impact on clinical outcomes [16, 41, 80]. For many years, the prevailing assumption was that DCI is primarily driven by large-vessel vasospasm. Consequently, most diagnostic efforts have traditionally focused on detecting vasospasm. Although the precise pathophysiology of DCI remains incompletely understood, it is now recognized to involve a range of interrelated mechanisms, including large-vessel vasospasm, microcirculatory dysfunction, inflammation, coagulation disturbances, and neuroelectric disruption [16, 20].

Advances in neuroimaging and neurophysiological monitoring have supported the multifactorial nature of DCI. This growing body of evidence has further substantiated diagnostic modalities that assess not only large-vessel dynamics but also microvascular function, neuroelectric activity, and metabolic state [15, 16, 20, 41, 81]. A broad range of diagnostic modalities has emerged, each targeting different aspects of the complex pathophysiology underlying DCI. These include neurophysiological techniques included in this review, such as transcranial Doppler (TCD), electroencephalography (EEG), and near-infrared spectroscopy (NIRS) [27, 78, 86].

Accurately predicting and timely detection of the risk of DCI is essential for enabling preventive interventions. Most diagnostic approaches target one or more specific components of the underlying pathophysiology. Research involving these modalities may not only enhance our understanding of the underlying mechanisms of DCI but also contribute to the development and optimization of therapeutic strategies aimed at reducing its incidence and improving clinical outcomes. Additionally, such research may help prevent unnecessary diagnostics, treatments, and prolonged hospitalizations.

Recent systematic reviews have assessed noninvasive bedside monitoring for DCI in aSAH, including meta-analyses on NIRS neuromonitoring, TCD-derived measures, and TCD and EEG. These reviews underscore heterogeneity in how signal-derived metrics and thresholds are defined and reported across studies [4, 11, 61].

However, they are largely modality-specific and meta-analytic, and a broader overview is needed of what each modality measures, how signal-derived metrics are generated, how outcomes are defined, and how findings relate to the timing of DCI.

### Objectives

A scoping review was conducted to systematically and critically appraise the existing literature on diagnostic neurophysiological modalities in aSAH related to the detection of DCI. The review specifically aims to provide a comprehensive overview of diagnostic neurophysiological modalities, the analytic methods applied within these modalities, their reported diagnostic accuracy, and their temporal aspects for DCI. Ultimately, the findings are aimed at supporting clinical applicability and guiding future research directions.

A preliminary search of MEDLINE, the Cochrane Database of Systematic Reviews, and the Joanna Briggs Institute (JBI) Evidence Synthesis identified several recent modality-specific meta-analyses, a scoping review synthesizing analytical strategies and temporal aspects across modalities was lacking.

## Methods

This scoping review was conducted in accordance with the Preferred Reporting Items for Systematic Reviews and Meta-Analyses extension for Scoping Reviews (PRISMA-ScR) [72].

### Eligibility criteria

We included articles published in English between 2004 and January 8, 2025, that reported on the diagnostic accuracy of neurophysiological modalities for identifying patients who subsequently developed DCI or closely related outcomes in aSAH. We excluded studies that primarily involved patients under 18 years of age, included fewer than 10 patients, were published in languages other than English, were case reports, reviews, or conference abstracts. Study selection criteria are summarized in Table 1, using the PICOS framework (Population, Intervention, Comparison, Outcomes, Study Design).

**Table 1:** Scope of the literature review in the PICOS framework

Criteria	Definition
Population	Patient with aSAH, aged ≥ 18 years
Interventions	Not applicable
Comparison	Diagnostic modality
Outcomes	DCI or DCI-related outcomes
Study Design	Prospective and retrospective diagnostic accuracy studies with ≥ 10 patients

*aSAH* aneurysmal subarachnoid hemorrhage, *DCI* delayed cerebral ischemia

The following definitions for DCI and associated outcomes were accepted: delayed cerebral infarction on imaging; delayed cerebral infarction with clinical symptoms; radiological and/or clinical criteria; symptomatic cerebral vasospasm; clinical deterioration attributed to DCI; clinical symptoms and vasospasm. All definitions excluded other potential causes of clinical deterioration. Eligible studies were expected to be broadly aligned with the Standards for Reporting Diagnostic Accuracy Studies (STARD) guidelines [7]. Diagnostic accuracy should be reported in terms of area under the area under the receiver operating characteristic curve (AUC) and/or sensitivity and specificity. While not mandatory, reporting of positive predictive value (PPV) and negative predictive value (NPV) was considered advantageous.

Studies identified through the search strategy are presented in a flowchart according to the PRISMA guidelines.

### Sources and search strategy

The following electronic bibliographic databases were systematically searched: PubMed, Embase (Elsevier) and Web of Science. No date or language restrictions were applied. In addition to the systematic database searches Google Scholar was searched as an additional source; the first 100 items were screened for relevance and added value. Unpublished studies were not sought, as only published studies were included.

We searched electronic bibliographic databases for studies on aneurysmal subarachnoid hemorrhage, combining terms for the condition, diagnostic modalities (including neuroimaging, neuromonitoring, and EEG), outcomes (delayed cerebral ischemia or symptomatic vasospasm), and measures of diagnostic performance (e.g., sensitivity, specificity, predictive value). Animal studies were excluded.

Keywords for the search were initially identified using a list of 41 potentially relevant articles found via orientation in the literature. This list was further used to develop and validate the search strategies. The complete search strategies for PubMed, Embase, and Web of Science were provided in Supplement S1.

The final search results were exported to EndNote, and duplicates were removed using Bramer’s method (a sequential procedure applying repeated “Find Duplicates” on field combinations such as, author–year–title; title; author–year–pages; DOI; and journal), as implemented by the information specialist [8]. The literature was initially searched using the predefined strategy on April 20, 2024, and updated on January 8, 2025.

### Selection of sources of evidence

After removing duplicates, the search results were imported into Rayyan for title and abstract screening [45]. Two authors independently screened all records to determine eligibility for full-text review based on the predefined inclusion criteria. Discrepancies in eligibility assessments were resolved through consensus. Subsequently, one author retrieved and assessed the full-text articles to determine final inclusion.

### Data charting process

Data were extracted from the included studies by one author using a predefined data charting template. This template captured key study characteristics, including first author, year of publication, diagnostic modality used, analytical approach, sample size, and reported diagnostic performance metrics (sensitivity, specificity, PPV, negative predictive value, and AUC). A second author independently verified the extracted data to ensure accuracy and consistency.

### Appraisal of individual sources of evidence

Given the substantial heterogeneity across studies in neurophysiological techniques, analytic approaches, and definitions of DCI and DCI-related outcomes, we aimed to provide a comprehensive overview of neurophysiological modalities, including both well-established and less commonly investigated approaches. As is typical for scoping reviews, which are primarily intended to map the breadth and scope of a research field, no formal grading of evidence or risk of bias assessment was performed. Data items and synthesis of results.

Studies were grouped according to diagnostic modality. For each modality, a general description is provided, including its theoretical framework and underlying technology. This is followed by a detailed account of the specific analytical methods applied and the corresponding results regarding diagnostic accuracy, with attention to temporal aspects and DCI outcome definitions. The key findings are substantiated by the extracted data and presented in structured tables, followed by a comprehensive synthesis and interpretation of the results.

### Classification of analytic approaches

For clarity and to facilitate comparability across studies, we classified each analysis into one of four categories:

- Qualitative: Visual interpretation or descriptive detection without quantitative analysis and without any predefined or cohort-derived numeric thresholds.
- Semi-quantitative: use of fixed thresholds or ordinal categories adopted from prior literature or clinical convention, rather than optimized in the current dataset (e.g., none/mild/moderate/severe).
- Quantitative: Continuous metrics with study-specific thresholds (ROC-optimized cutoffs) or evaluation of performance on continuous scales (AUC).
- Models: Models that combine 2 or more predictors (within or across modalities and/or clinical variables), producing a classification of DCI risk.

If a study reported multiple analytic approaches, we classified and extracted each approach separately. Therefore, a single study may appear in more than one category. Where applicable, we also highlight the best-performing approach.

### DCI outcome definitions

To clarify the outcome definitions used across studies, DCI-related outcomes were categorized into the following groups:

i. Delayed cerebral infarction on imaging
ii. Delayed cerebral infarction with clinical symptoms
iii. Radiological and/or clinical criteria
iv. Clinical deterioration attributed to DCI
v. Clinical symptoms and vasospasm

Across all categories, definitions required exclusion of alternative causes for the observed clinical and/or radiological findings.

## Results

### Selection of sources of evidence

**Fig. 1:**
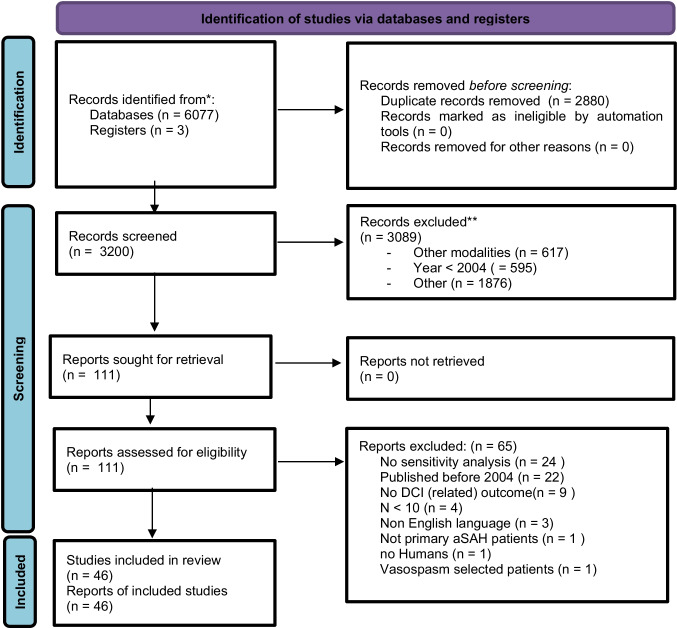
PRISMA flow diagram

Through our systematic search strategy, we identified 3,200 records after deduplication (Fig. 1). Two authors independently screened the titles and abstracts of these records. Of the 3,200 records, 111 were selected for full-text review and subsequently categorized by modality. Following full-text assessment, 46 studies were included in this review (Table 2).

**Table 2:** Summary of study characteristics, sample size, and delayed cerebral ischemia incidence

Modality	Retrospective	Prospective	*N* total	*N* DCI
Transcranial Doppler	16	10	3142	690 (22%)
Near-infrared spectroscopy	2	4	613	270 (44%)
Electroencephalography	7	7	775	395 (51%)

*DCI* delayed cerebral ischemia

### Characteristics of the search

The search identified publications between 1964 and 2025, with a steady annual increase in the number of articles since the early 1990s. The highest number of publications was observed in 2022 (208 papers retrieved). Ultimately, only studies published from 2004 onward were included, resulting in the exclusion of an additional 595 studies (Supplementary Table S3). Publications were distributed across 156 different journals, with the highest numbers found in Neurosurgery (*n* = 175), Stroke (*n* = 165), Journal of Neurosurgery (*n* = 163), Neurocritical Care (*n* = 127), and World Neurosurgery (*n* = 11; Supplementary Table S4).

Our systematic search was initially designed to capture all potentially relevant diagnostic modalities, which identified 46 distinct diagnostic modalities. To limit the scope, radiological modalities such as non-contrast computed tomography (CT), computed tomography angiography (CTA), computed tomography perfusion (CTP), digital subtraction angiography (DSA), and magnetic resonance imaging (MRI) were excluded, since these modalities provide intermittent assessments rather than continuous monitoring. Likewise, biomarkers and modalities with nuclear tracers, such as single-photon emission computed tomography (SPECT) and xenon-enhanced computed tomography (Xe-CT), were also excluded. Moreover, nuclear imaging is less suitable for routine patient care. Invasive techniques such as intracranial pressure monitoring and cerebral microdialysis do allow continuous measurements but were excluded because of their invasiveness, limited availability, and higher threshold for use (Supplementary Table S2). Therefore, all in all, this review focuses on three non-invasive neurophysiological techniques, TCD, EEG, and NIRS. These noninvasive modalities allow for continuous bedside monitoring and enable nearly real-time assessment without procedural risks, support timely therapeutic adjustments, and may lead to decisions regarding targeted diagnostic procedures.

A total of 111 studies on neurophysiological techniques were selected for full-text screening. This included 26 of 75 studies on TCD, 14 of 27 studies on EEG, and 6 of 9 studies on NIRS (Supplementary Table S2).

### Characteristics of included sources of evidence

**Fig. 2:**
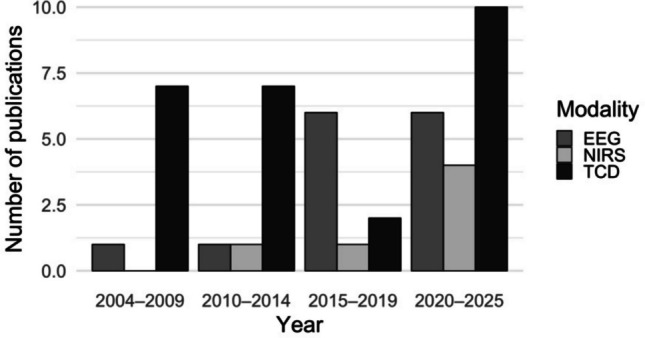
Number of included publications per modality. EEG indicates electroencephalography; NIRS, near-infrared spectroscopy; and TCD transcranial Doppler

Of the 46 studies included in this review, 25 studies were retrospective and 21 prospective. Of 26 TCD studies, including 3,142 patients, 690 (22%) developed DCI. The 6 NIRS studies included 613 patients, with 270 (44%) developing DCI. For EEG, 14 studies were included, comprising 775 patients, of whom 395 (51%) developed DCI. Over the past 20 years, a steady increase has been observed in the number of studies eligible for inclusion in this review (Fig. 2). See Table 2 and Supplementary Table S5 for further descriptive details of the included studies.

## Transcranial Doppler: Synthesis of results

Through title and abstract screening, we identified 75 papers utilizing TCD; following full-text screening, 26 papers were included. TCD emerged as the second most used screening tool in our literature search, after non-contrast CT (Supplementary Table S7).

### Technical aspects of transcranial Doppler

TCD is used to noninvasively assess intracranial blood flow velocities.TCD studies showed good consistency across the various studies. A key difference lies in whether only TCD, also known as non-duplex TCD, is used or whether it is combined with B-mode imaging, also referred to as transcranial color-coded duplex (TCCD). Typically, a 2 MHz probe is employed, as it allows measurements through bone [40, 50].

In the studies included in this review, non-duplex TCD is the most employed method for monitoring. In non-duplex TCD, arteries are identified based on the insonated bone window, probe orientation, and measurement depth [40, 50]. In addition to these conventional TCD methods, we identified one study that employed ultrasound perfusion imaging to predict DCI [19], and another study that used emboli detection [56]. Emboli reflect a prothrombotic state leading to (micro)thrombi in the cerebral microcirculation; they typically arise within the same vascular territory as the ischemic lesions [56]. All studies focusing on blood flow velocities measured the middle cerebral artery (MCA). In addition, the extracranial internal carotid artery (ICA) was frequently assessed to calculate the Lindegaard ratio. There is considerable variation across studies in terms of whether the anterior cerebral artery (ACA), posterior cerebral artery (PCA), basilar artery (BA), and vertebral arteries (VA) are included in measurements. Almost all studies assess the mean flow velocity (MFV) [2, 9, 10, 19, 23, 29, 32, 34, 39, 42, 51, 52, 56, 62, 66, 69, 76, 84, 85, 87]. The peak systolic velocity (PSV), end-diastolic velocity (EDV), and pulsatility index were infrequently included in the analyses; however, some studies used these measurements in addition to the mean flow velocity (MFV) [23, 52].

TCD measurements typically commence between days 0 and 3 and continue until usually up to approximately day 14. The standard practice involves daily or every other day measurements, with more frequent assessments if clinical changes warrant [52, 68].

### Analysis of transcranial Doppler signals

Most TCD studies assessed flow velocity as a predictor for vasospasm and subsequent DCI risk. The MCA-MFV was the most frequently used variable, with cutoffs ranging from ≥ 120–125 cm/s for mild to > 175–200 cm/s for severe vasospasm, often combined with the Lindegaard ratio [9, 29, 51, 76, 77]. Some studies evaluated relative changes in MFV [32, 42], the pulsatility index [14, 52], or other velocity measures such as EDV and PSV, though these provided no consistent incremental value [13]. Alternative approaches included emboli detection [56], ultrasound perfusion imaging [19], or physiological models such as the cerebral arterial time constant [74]. Recent work has also explored multimodal approaches, integrating TCD with non-vascular markers such as serum melatonin [67] or qEEG features [14].

Representative thresholds and analytic approaches are summarized in Table 3.

**Table 3:** Transcranial Doppler based analysis for detection of delayed cerebral ischemia in aneurysmal subarachnoid hemorrhage

Analysis	Authors	Diagnostic accuracy in representative studies	Outcome categories in representative studies
Qualitative			
Emboli detection in the MCA, 30 min, 5 days/week	Romano [56]	*n* = 40: Sens 0.63 (95% CI 0.38–0.84), Spec 0.86 (95% CI 0.64–0.97), PPV 0.80, NPV 0.72 [56]	**IV**. Clinical deterioration attributed to DCI
Semi quantitative			
Based on vasospasm cutoff value: MCA-MFV > 120 cm/s	Carrera [9]; Lee [29]; Rabinstein [51]; Snider [66]; van der Harst [76] (2019); Wang [84] Wang [85] Pham [49] Westermaier [87]	*n* = 441: Sens 0.63, Spec 0.52, PPV 0.26, NPV 0.84 [9]	**III.** Radiological and/or clinical criteria
Moderate vasospasm: MCA-MFV 150–200 cm/s, severe > 180–200 cm/s, Lindegaard ratio > 3 (mild), > 6 (severe)	Lee [29]; Snider [66];van der Harst [76]; Ognard [44]	*n* = 262: Sens 0.56, Spec 0.69, PPV 0.17, NPV 0.93 [66]	**I.** Delayed cerebral infarction on imaging
Vasospasm: MCA-MFV > 120; BA-MFV > 85–120; ACA-MFV > 80–120 cm/s; Sloan ratio > 4 (ACA/ICA)	van der Harst [76]; Snider [66]; Miller [34]; Pham [49]	*n* = 38: Sens 0.58 (95% CI 0.32–0.82), Spec 0.50 (95% CI 0.37–0.62), PPV 0.37, NPV 0.71 [49]	**I.** Delayed cerebral infarction on imaging
Change in MCA-MFV over time, e.g., ≥ 50 cm/s increase in 2 days or 2 × baseline	Malhotra [32]; Naval [42]; Han [23]; Chang [10]; Ognard [44]	*n* = 95: AUC 0.65, Δ > 8.9 cm/s: Sens 0.65, Spec 0.70, PPV 0.46, NPV 0.84; Δ > 45.2 cm/s: Sens 0.60, Spec 0.59, PPV 0.40, NPV 0.77, AUC 0.60 [10]	**I.** Delayed cerebral infarction on imaging
Composite severity score based on vasospasm in MCA, ACA, and PCA	van der Harst (2024) [77]	*n* = 621: AUC 0.64 (95% CI 0.56–0.71), cutoff ≥ 1 (days 2–5): Sens 0.53, Spec 0.74 [77]	**III**. Radiological and/or clinical criteria
Quantitative			
MCA-MFV with calculated cutoff values	Malhotra [32]; Scherle Matamoros [62]; Toi [69]; Nakae [39]; Carrera [9]; Dai [14]	*n* = 211: AUC 0.80 (95% CI 0.74–0.88); cutoff > 175 cm/s: Sens 0.75, Spec 0.84, PPV 0.58, NPV 0.92 [32]	**V.** Clinical symptoms and vasospasm
MFV, PSV, EDV of MCA and ratios with the external ICA	Dabecco [13]	*n* = 103: AUCs – MFV 0.68, PSV 0.68, EDV 0.69, MCA/Ext. ICA: MFV 0.62, PSV 0.65, EDV 0.64 [13]	**IV**. Clinical deterioration attributed to DCI
Pulsatility index = (PSV – EDV)/MFV	Rajajee [52]	*n* = 81: AUC 0.79 (95% CI 0.69–0.88); cutoff 0.58: Sens 0.71, Spec 0.88 (Rajajee 2012)	**V**. Clinical symptoms and vasospasm
Models			
Ultrasound perfusion: TTP, MTT in multilayer classification model	Fung [19]	*n* = 27: Sens 0.93, Spec 0.91	**IV**. Clinical deterioration attributed to DCI
MCA-MFV combined with serum melatonin levels	Su [67]	*n* = 120: AUC 0.92 (95% CI 0.85–0.95), Sens 0.91, Spec 0.95 [67]	**V**. Clinical symptoms and vasospasm
MCA-MFV combined with alpha/delta ratio by qEEG	Dai [14]	*n* = 105: AUC 0.96 (95% CI 0.90–0.99); Sens 0.91, Spec 0.95 [14]	**V.** Clinical symptoms and vasospasm
Cerebral arterial time constant (τ) from ABP and MCA-MFV waveforms	Uryga [74]	*n* = 71: AUC 0.75, cutoff: MCA-MFV > 86 cm/s & τ < 247 ms; Sens 0.47, Spec 0.83, PPV 0.47, NPV 0.83 [74]	**III**. Radiological and/or clinical criteria

*MCA* middle cerebral artery, *Sens* sensitivity, *CI* confidence interval, *Spec* specificity, *PPV* positive predictive value, *NPV* negative predictive value, *MFV* mean flow velocity, *BA* basilar artery, *ACA* anterior cerebral artery, *ICA* internal carotid artery, *AUC* area under the receiver operating characteristic curve, *PCA* posterior cerebral artery, *PSV* peak systolic velocity, *EDV* end-diastolic velocity, *Ext. ICA* external internal carotid artery, *TTP* time to peak, *MTT* mean transit time, *qEEG* quantitative electroencephalogram, *τ* cerebral arterial time constant

### Diagnostic accuracy of transcranial Doppler in predicting delayed cerebral ischemia qualitative analysis

In qualitative analyses, one study investigated TCD using emboli detection and reported moderate sensitivity but relatively high specificity [56].

In semi-quantitative analyses, the majority of studies applied fixed vasospasm thresholds, typically MCA-MFV ≥ 120—200 cm/s and Lindegaard ratio > 3—6. These approaches yielded modest accuracy, with AUC values around 0.60—0.65 and sensitivity and specificity in the 0.50–0.70 range [9, 66, 76]. Smaller series suggested higher accuracy when relative MFV increases were evaluated, though larger studies did not confirm this [32, 42].

In quantitative analyses, studies deriving ROC-based cutoffs generally reported better performance, with AUC values up to 0.80–0.81 and higher, and more balanced sensitivity and specificity [32, 39, 69]. Additional indices, such as pulsatility index [52] or MCA velocity ratios, showed variable results but no consistent advantage over MFV [39].

When using model-based approaches multivariable approaches achieved the highest diagnostic accuracy. Ultrasound perfusion imaging reached high sensitivity and specificity, [19] while models integrating MFV with serum melatonin [67] or qEEG-derived alpha/delta ratio reached AUC values up to 0.96 [14]. The cerebral arterial time constant combined with MFV also showed predictive value, though less strongly [74].

A complete overview of cutoff values, diagnostic accuracy metrics, and representative studies is provided in Table 3.

### Temporal relationship between transcranial Doppler and delayed cerebral ischemia onset

In most TCD studies, mean flow velocities peaked on days 5–8, when TCD generally performed best for predicting DCI. The interval between abnormal TCD measurements and DCI onset was rarely reported [9, 10, 34, 39, 42, 52, 66, 69, 85]. Where DCI onset was reported, it occurred early and rose sharply between days 3–7, [9, 39, 69] with mean onset around days 7–8 [42, 52]. A study, using a radiological definition of DCI reported a median onset on day 8 (IQR 6–11) but did not specify imaging frequency. TCD criteria predicted DCI best on days 4–5 in the same study [66]. Prediction of DCI within ≤ 24 h was reported when TCD was combined with melatonin, and in another study when it was combined with qEEG [14, 67]. Beyond classic vasospasm thresholds, approaches using nontraditional parameters or composite scores showed useful prediction in the 2–5 day window, [32, 52, 77] and even within 3 days of the ictus [62]. In one study, the median time from prediction to DCI onset was 1 day (IQR 0–3) [32]. TCD-detected microembolic signals occurred on average at 6.7 days (range 2–14) and were observed from 4 days before to 9 days after DCI onset, indicating ongoing microembolic activity [56]. In TCD studies, outcome definitions fell into several categories relevant to interpreting timing relative to DCI onset, including clinical deterioration attributed to DCI (26.9%), delayed cerebral infarction on imaging, radiological and/or clinical criteria, and clinical symptoms and vasospasm (each 23.1%), and delayed cerebral infarction with clinical symptoms (3.8%; Supplementary Table S5 and S6).

### Synthesis and interpretation of transcranial Doppler in predicting delayed cerebral ischemia

TCD is a long-established and widely applied technique, also used to predict the development of DCI in aSAH patients. While there are no major differences in TCD ultrasound equipment itself, factors such as operator expertise, measurement timing, and measurement duration substantially influence the results.

The predictive performance also depends on the type of variable analyzed. Most studies focus on flow velocity analysis, particularly of the MCA, but several studies have also examined flow velocities in other vessels, such as the ACA, PCA, BA, VA, and ICA. Flow velocities can be measured using MFV, PSV, EDV, pulsatility index, and various ratios, or by assessing dynamic changes over time. However, the studies show no consistent advantage for any specific variable, and the reported diagnostic performance and cutoff values vary widely across studies. Overall, most studies report a predictive value for TCD that ranges from limited to substantial when analyzing flow velocities (Table 3). In addition to flow velocity analysis, emboli detection has also demonstrated predictive potential [56], and ultrasound perfusion imaging, a relatively new technique, has shown promising results [19]. Combining TCD-derived variables into predictive models [19, 76] or integrating TCD data with other clinical or physiological variables appears to enhance the predictive value of TCD (Table 3) [14, 67, 74]. In most studies, flow velocities peaked during days 5–8, corresponding to the typical DCI window, and abnormal values often preceded clinical deterioration by 1–3 days [9, 10, 32, 34, 39, 42, 52, 56, 66, 69, 85]. These temporal findings should, however, be interpreted in light of the heterogeneity in outcome definitions used across studies (Supplementary Table S5).

In conclusion, although MCA-MFV remains the most frequently used variable, and there appears to be some consensus regarding TCD-based vasospasm thresholds, no clear consensus exists on the optimal cutoff values for DCI prediction. The research field continues to show considerable heterogeneity approaches with respect to the variables analyzed and cutoff selection. Nevertheless, TCD consistently demonstrates predictive value across a variety of approaches and models.

## Near-infrared spectroscopy: Synthesis of results

By title abstract screening, nine articles were eligible for full-text reading. Six articles were finally included in this review (See Supplementary Table S8). Of the articles assessed in full text, the earliest studies (published in 2007, 2008, and 2010) were excluded due to their descriptive nature, small sample size (*n* < 10), and lack of sensitivity analysis. The first included study, published in 2014, highlights that NIRS is a relatively novel technique that has only recently been applied in the context of aSAH.

### Technical aspects of near-infrared spectroscopy

NIRS can be used to measure brain oxygenation [18]. Optodes are placed on the skull to measure regional cerebral oxygen saturation (rSO₂) in brain tissue. The optodes are typically positioned on the forehead, measuring a part of the frontal cortex vascularized by the watershed border zones between the ACA and MCA [38, 47, 79].

NIRS measures the absorption of near-infrared (NIR) light (700–1000 nm), from which rSO₂ is calculated based on the absorption spectra [18, 38, 79].

The included studies primarily used NIRS signals related to oxygenated and deoxygenated hemoglobin, from which rSO₂ is derived. NIRS-derived rSO₂ reflects the combined venous and arterial oxygen saturation of hemoglobin in brain tissue [18, 38]. Additionally, several extracranial factors may act as confounders, including hemoglobin from a subdural hematoma, skull thickness, and the surface area of cerebrospinal fluid (CSF). Given these confounding factors, NIRS is often recommended as a trend-monitoring tool, comparing with individual baseline measurements rather than absolute values [38].

### Analysis of near-infrared spectroscopy signals

Most studies used INVOS continuous-wave (CW) oximeters [47, 48, 79, 92]. One study applied the MNIR-P100, for which no technical specifications were available, [30] and another did not specify the device [91]. All analyzed rSO₂, either quantitatively with ROC-derived cutoffs, [79, 91] semiquantitatively with predefined thresholds, [92] by analyzing relative decreases over time [47, 48], or via correlation between rSO₂ and MAP [30]. Representative analytic approaches are summarized in Table 4.

**Table 4:** Near-infrared spectroscopy based analysis for detection of delayed cerebral ischemia in aneurysmal subarachnoid hemorrhage

Analysis	Authors	Diagnostic accuracy in representative studies	Outcome categories in representative studies
Semi quantitative			
rSO₂ value below 50 for at least 30 min was used to indicate cerebral desaturation. Measurements were performed continuously between day 5 and day 10 after ictus	Yousef [92]	*n* = 163: Sens 0.66 (95%CI 0.55—0.75), Spec 0.57 (95%CI 0.45—0.69), PPV 0.67 (95%CI 0.60—0.73), NPV 0.56 (95%CI 0.47—0.64) [92]	**V.** Clinical symptoms and vasospasm
Decrease in rSO₂ was monitored through continuous recordings over a 14-day period following the ictus. The decline was calculated using the first day as the reference, and compared to the daily average rSO₂ values	Park (2020) [48]; Park (2021) [47]	*n* = 52: AUC 0.87; cutoff Reduction rSO2 > 12.7%, Sens 0.94 (95%CI 0.73—0.100), Spec 0.71 (95%CI 0.53—0.85) [48]	**V**. Clinical symptoms and vasospasm
Quantitative			
Bilateral rSO₂ values were assessed within 48 to 72 h after surgery. The duration of rSO₂ monitoring was not explicitly reported	Yang [91]	*n* = 252: AUC 0.93 (95%CI 0.90—0.92); cutoff left > 68.5%, right 69.5%, Sens 0.89, Spec 0.84 [91]	**IV**. Clinical deterioration attributed to DCI
rSO₂ measurements were typically repeated one to three times, depending on the clinical condition. The mean and standard deviation were calculated across the entire measurement period. measurement	van der Harst [79]	*n* = 41: AUC 0.77 (95%CI 0.62—0.92); cutoff > 65%, Sens 1.0, Spec 0.45, PPV 0.43, NPV 1.0 [79]	**III.** Radiological and/or clinical criteria
Models			
Pearson correlation coefficient (R) between rSO₂ and mean arterial pressure (MAP) was used to evaluate cerebral autoregulation (CA). An index of R value (|R|) ≥ 0.5 was defined as impaired CA, while index|R|< 0.05 was defined as intact CA	Liu [30]	*n* = 81: Sens 0.87 (95%CI 0.73—0.94), Spec 0.67 (95%CI 0.49—0.81), PPV 0.76 (95%CI 0.67—0.84), NPV 0.80 (95%CI 0.65—0.90) [30]	**IV**. Clinical deterioration attributed to DCI

*rSO₂* regional cerebral oxygen saturation, *Sens* sensitivity, *CI* confidence interval, *Spec* specificity, *PPV* positive predictive value, *NPV* negative predictive value, *AUC* area under the receiver operating characteristic curve

### Diagnostic accuracy of near-infrared spectroscopy in predicting delayed cerebral ischemia

Across studies, NIRS demonstrated fair to good predictive accuracy, with AUC values mostly in the 0.77–0.93 range [79, 91]. Absolute cutoff values, typically between 65–70%, [79, 91] and trend-based analyses of rSO₂ decline [47, 48] performed comparably, whereas predefined cutoff values showed lower accuracy [92]. Sensitivity was generally high across studies, but specificity was more variable, with some studies reporting lower values [79, 92]. A model combining rSO₂ and MAP did not outperform rSO₂ alone [30]. A complete overview of cutoff values, diagnostic accuracy metrics, and representative studies is provided in Table 4.

### Temporal relationship between near-infrared spectroscopy in predicting delayed cerebral ischemia

NIRS studies included in this review provided limited quantitative data on the intervals between NIRS abnormalities and DCI onset. One study found that DCI occurred mainly on days 5–11, peaking at days 7–8, with NIRS abnormalities preceding onset by 0–3 days [48]. Across these studies, NIRS rSO₂ time courses for patients with subsequent DCI began to separate from those without DCI around day 3, falling within the same time window as the earliest clinical DCI cases [30, 47, 48, 91]**.** In NIRS studies, outcome definitions included several categories relevant to interpreting timing relative to DCI onset, including clinical symptoms and vasospasm (50.0%), clinical deterioration attributed to DCI (33.3%), and radiological and/or clinical criteria (16.7%; Supplementary Table S5 and S6).

### Synthesis and interpretation of near-infrared spectroscopy in predicting delayed cerebral ischemia

Although NIRS is not yet widely or routinely used for predicting DCI, the available studies suggest that it offers added value in this context. NIRS evaluates a fundamentally different pathophysiological process compared to TCD. While TCD assesses large vessel flow, NIRS reflects the final metabolic consequence of cerebral perfusion, capturing downstream effects, particularly in the microvasculature. NIRS abnormalities generally appeared early, typically around days 3 and up to several days before clinical DCI onset, indicating that rSO₂ trends may provide an early metabolic correlate of evolving ischemia [30, 47, 48, 91]. However, interpretation of these temporal findings is complicated by variation in outcome definitions across studies (Supplementary Table S5).

There are several commercially available NIRS devices based on different underlying technologies, used to measure rSO₂ and other variables either in absolute or relative terms. To date, no studies have investigated the more complex FD-NIRS or TD-NIRS techniques in the prediction of DCI. As far as we could determine, all included studies were based on CW-NIRS, and the rSO₂ variable was used exclusively for analysis. Different versions of the INVOS cerebral somatic oximeter were the most employed system.

Studies that determined their own cutoff values using ROC analysis demonstrated the highest diagnostic performance. Interestingly, the optimal cutoff values for absolute rSO₂ measurements were relatively consistent across studies, typically ranging from 65 to 70% [79, 91]. Theoretically, analyses based on a decrease in rSO₂ may perform better, as baseline rSO₂ levels can vary between patients. However, the outcomes of these trend-based approaches were comparable to those based on absolute values [47, 48].

The use of predefined fixed criteria appears less effective. This may be due to the fact that such thresholds were likely derived from different patient populations, and the applied cutoff was substantially lower, which may explain the reduced sensitivity [92]. The model that combined rSO₂ and MAP did not show improved performance over rSO₂ alone. However, it would have been informative how rSO₂ performed independently in their dataset, and what optimal rSO₂ threshold they would have identified [30].

In conclusion, all included studies measured rSO₂ in the frontal region. These NIRS-based measurements appear to provide a clinically meaningful prediction of DCI. No significant differences have been observed between trend-based and absolute-value approaches. However, cutoff values may need to be device and population-specific, and potentially even calibrated per clinical center. It is likely also valuable to extend measurement beyond the frontal region by using multiple sensors placed across different vascular territories [18, 71, 89].

The studies included in this review demonstrate that, when appropriately calibrated, rSO₂ can achieve promising diagnostic accuracy.

## Electroencephalography: Synthesis of results 

By title and abstract screening, 26 articles were eligible for full-text reading, of which 14 were included in this review. The primary reason for exclusion was the absence of a sensitivity analysis (*n* = 5) (Supplementary Table S9). This represents a substantially higher number of included studies compared to the six identified for NIRS, but still fewer than the 26 studies included for TCD, which remains the most extensively studied neurophysiological screening modality.

### Technical aspects of electroencephalography

Studies included in this review utilized noninvasive scalp EEG, studies using only invasive electrocorticography were excluded. All EEG studies applied the international 10/20 EEG system, although not all used the full set of electrodes [3, 12, 21, 22, 37, 88]. In addition to classifying findings by hemisphere, several studies also examined the relationship between vascular territories and the spatial location of EEG electrodes [3, 12, 65, 88, 93]. Sampling rates and filter settings were generally comparable across studies [3, 12, 54, 93].

### Analysis of electroencephalography signals

Before analysis, EEG data were filtered and preprocessed to reduce artifacts, using manual or automated approaches [3, 12, 14, 21, 26, 37, 59, 88]. In studies based solely on visual interpretation, artifact rejection was often implicit rather than explicitly reported [57, 63, 65].

Quantitative electroencephalography (qEEG), particularly power spectral analysis of standard frequency bands, was the most used technique in EEG studies. Derived ratios such as the alpha/delta ratio were frequently investigated [3, 12, 37, 59]. Other analytic strategies included coherence measures, the brain symmetry index, alpha variability, and normalization against baseline recordings [3, 37, 88]. One study focused on epileptiform discharges instead of qEEG features [26]. Semiquantitative analyses typically employ predefined qEEG criteria, such as percentage declines in alpha power [21, 37, 88]. Qualitative assessment involved expert visual interpretation of EEG patterns and, in some studies, application of alarm criteria [57, 63, 65]. Several models further integrated qEEG features with other variables, including machine-learning classifiers, [93] composite indices with clinical data, [54] and combined qEEG and TCD measures [14]. Representative analytic approaches are summarized in Table 5.

**Table 5:** Electroencephalography based analysis for detection of delayed cerebral ischemia in aneurysmal subarachnoid hemorrhage

Analysis	Authors	Diagnostic accuracy in representative studies	Outcome categories in representative studies
Qualitative			
Alarm criteria: focal slowing or dropout of fast activity, decreasing relative alpha variability, decreasing alpha-to-delta power ratio, and increasing epileptiform abnormalities	Rosenthal [57]	*n* = 103: any EEG alarm: Sens, 0.96, Spec 0.80, PPV 0.83, NPV 0.95 [57]	**III**. Radiological and/or clinical criteria
cEEG findings were considered positive if the final report of the attending epileptologist indicated vasospasm	Scherschinski [63]	*n* = 71: Sens 0.11(95%CI 031—0.26), Spec 0.83 (95%CI 0.66—0.93) [63]	**III**. Radiological and/or clinical criteria
New or worsening epileptiform abnormalities and new background activity deterioration	Sivakumar [65]	Epileptiform abnormalities: Sens 0.83, Spec 0.43, PPV 0.77, NPV 0.42 Background deterioration: Sens 0.92, Spec 0.50, PPV 0.81, NPV 0.93 [65]	**III**. Radiological and/or clinical criteria
Quantitative			
Alpha-theta/delta ratio decrease	Balança [3]	*n* = 15: regional 30% decrease outlasting 3.7 h; Sens 1.0, Spec 0.89 [3]	**I.** Delayed cerebral infarction on imaging
Absolute power (total, alpha, delta, beta, fast), relative power (alpha/total, delta/total, alpha/delta, fast/delta), coherence (alpha power, delta power), and average frequency	Claassen [12]; Dai [14]	*n* = 34: Relative alpa/delta power ratio AUC 0.83 (95%CI 0.75—0.91) 6 consecutive post-stimulation recordings with a 10% decrease in alpha/delta ratio from baseline; Sens 1.0, Spec 0.76, PPV 0.60, NPV 1.0 Any single measurement with a 50% decrease of the alpha/delta ratio (Sens 0.89, Spec 0.84, PPV 0.67, NPV 0.96 [12]	**V**. Clinical symptoms and vasospasm
Absolute power (alpha, beta, delta, theta) and alpha/delta power ratio	Gollwitzer [22]; Mueller [37]	*n* = 12: Alpha: AUC 0.66; cutoff power decrease 40%, duration 5 h, Sens 0.89, Spec 0.77 Beta: AUC 0.66; cutoff power decrease 50%, duration 3 h, Sens 0.78, Spec 0.69 Delta: AUC 0.52; cutoff power decrease 70%, duration 2 h, Sens 0.55, Spec 0.69 Theta: AUC 0.72; cutoff power decrease 40%, duration 6 h, Sens 0.89, Spec 0.77 Alpha/delta ratio: AUC 0.64; cutoff power decrease 70%, duration 2 h, Sens 0.55, Spec 0.85 [22]	**I.** Delayed cerebral infarction on imaging
The epileptiform discharge burden was calculated as the number of discharges (sporadic or periodic) detected per hour. Analyses were conducted for three temporal patterns: hourly epileptiform discharge burden following aSAH, cumulative epileptiform discharge burden following aSAH, and discharge burden aligned to the time of DCI	Kim [26]	*n* = 113: Hourly epileptiform discharges burden after aSAH (mean from day 3.5 to day 6): AUC range 0.61—0.62, Sens 0.60, Spec 0.70; Cumulative epileptiform discharges burden after SAH (up to day 4.8): AUC 0.61, Sens 0.66 Spec 0.60; Epileptiform discharge burden aligned to DCI onset (5 days prior to DCI): AUC 0.72; Sens 0.74; Spec 0.70 [73	**III.** Radiological and/or clinical criteria
Parameters of alpha power decline: qEEG event (alpha power decline of ≥ 40% lasting for ≥ 5 h compared to the baseline), maximum percentage of alpha power decrease in one channel, maximum hours of alpha power decrease ≥ 40% in one channel, and total (summed) hours of alpha power decrease ≥ 40% considering all channels	Mueller [37]	*n* = 24 Pre-defined EEG event: Sens 1.0, Spec 0.32; percentage alpha power decrease: AUC 0.77; cutoff 77%, Sens 0.56, Spec 0.88; maximum hours power decrease: AUC 0.78; cutoff 47 h, Sens 0.78, Spec 0.80; maximum hours power decrease: cutoff 158 h; AUC 0.82; Sens 0.78, Spec 0.84 [37]	**I.** Delayed cerebral infarction on imaging
Power spectral densities were estimated using Welch’s method, from which 12 qEEG features were calculated	Rots [59]	*n* = 20: Alpha/delta ratio and alpha variability; AUC 0.92 (95%CI 0.74—1.0) cutoff decrease of > 38%, Sens 1.0 Spec 0.83 alpha/delta ratio: AUC 0.90 (95%CI 0.71—1.0) alpha variability: AUC 0.73 (95%CI 0.43—1.0) [59]	**III**. Radiological and/or clinical criteria
Semi quantitative			
Alpha power decline of ≥ 40% lasting for ≥ 5 h	Gollwitzer [21]; Mueller [37]	*n* = 22: No signicant association between EEG events and ischemic lesions on imaging (P = 0.1) [21]	**I**. Delayed cerebral infarction on imaging
Automated methods: Alpha/delta ratio with a fixed baseline, a threshold of − 10%, and an alarm criterion of 2 consecutive alpha/delta ratio below baseline Relative alpha variability score (1—4) alarm is emitted for each 12 h in which there is a decrease in the score in two or more brain region	Wickering [88]	*n* = 95: Alpha/delta alarm: Sens 0.95, Spec 0.34 Relative alpha variability alarm: Sens 0.65, Spec 0.43 [88]	**III**. Radiological and/or clinical criteria
Models			
Nine quantitative EEG features were extracted: epileptiform discharge burden, Shannon entropy, delta (0.5–4 Hz), theta (4–7 Hz), alpha (8–15 Hz), beta (16–31 Hz), total (0.5–15 Hz) band power, alpha/delta ratio, and percent alpha variability. Combining all features in a random forrest model, which averages the prediction probabilities of successive models at six-hour intervals “max carry forward” model	Zheng [93]	*n* = 107: max carry forward model day 5; AUC 0.73 (95%CI 0.69—0.77), Sens 0.72, Spec 0.65 [93]	**III**. Radiological and/or clinical criteria
Composite alpha index is defined as the product of the standard deviation and mean alpha power Predictions were based on clinical data in combination with composite alpha index	Rathakrishnan [54]	*n* = 12: Clinical data and composite alpha index, Sens 0.67 (95%CI 0.45—0.83), Spec 0.73 (95%CI 0.65—0.79) [54]	**I.** Delayed cerebral infarction on imaging
Relative alpha/delta power ratio with MCA-MFV	Dai [14]	*n* = 105: AUC 0.96 (95%CI 0.90—0.99); cutoff not reported, Sens 0.91, Spec 0.95 [14]	**V**. Clinical symptoms and vasospasm

*Sens* sensitivity, *Spec* specificity, *PPV* positive predictive value, *NPV* negative predictive value, *AUC* area under the curve, *CI* confidence interval, *cEEG* continuous electroencephalography, *qEEG* quantitative electroencephalography, *DSA* digital subtraction angiography, *aSAH* aneurysmal subarachnoid hemorrhage, *DCI* delayed cerebral ischemia, *MCA* middle cerebral artery, *MFV* mean flow velocity

### Diagnostic accuracy of electroencephalography in predicting delayed cerebral ischemia

Overall, qEEG studies that applied quantitative analysis reported fair to excellent diagnostic accuracy, with the alpha/delta ratio consistently among the strongest predictors [12, 14, 59]. In addition to qEEG features, one quantitative study analyzed epileptiform discharge burden as a predictor of DCI. This analysis reported only moderate accuracy. [26] Semiquantitative approaches performed similarly, though variability in criteria resulted in a wide range of sensitivities and specificities [37, 88]. Qualitative studies showed heterogeneous results. One study using structured alarm criteria reported high accuracy, [57] whereas others demonstrated the expected trade-off between sensitivity and specificity [63, 65]. Among predictive models, the best performance was achieved by combining the qEEG alpha/delta ratio with TCD MFV [14]. In contrast, models based solely on qEEG features or combined with clinical data showed only moderate accuracy [54, 93]. A complete overview of cutoff values, diagnostic accuracy metrics, and representative studies is provided in Table 5.

### Temporal relationship between electroencephalography in predicting delayed cerebral ischemia

One EEG study reported that qEEG changes preceded clinical DCI by a median of 7 h (IQR − 11 to + 25) and CT changes by 44 h (IQR 14–117) [59]. Other studies reported that qEEG abnormalities preceded DCI by 2–4 days [22, 37, 57, 88] and were also observed to precede TCD-defined vasospasm [21, 22]. In EEG-monitored patients, DCI typically occurred around days 6–7 [57, 88]. Epileptiform discharge burden predicted DCI up to 5 days in advance [26], and a machine-learning model using multiple EEG features performed best when time-aligned to DCI rather than to the ictus, with optimal discrimination 2 days before onset. [93] In EEG studies, outcome definitions fell into several categories relevant to interpreting timing relative to DCI onset, including radiological and/or clinical criteria (57.1%), delayed cerebral infarction on imaging (35.7%), and clinical symptoms and vasospasm (7.1%; Supplementary Table S5 and S6).

### Synthesis and Interpretation of electroencephalography in predicting delayed cerebral ischemia

EEG is one of the oldest neurophysiological techniques and has been applied across various clinical domains [35, 58]. Since the 1990 s, it has increasingly been used to detect and predict the development of cerebral ischemia. EEG provides a direct reflection of neuronal activity at the cellular level [25]. In particular, qEEG reflects cortical hypoactivity and interhemispheric asymmetry, which are closely related to cerebral energy metabolism and functional status [14, 25]. These features have been used to anticipate impending ischemia, while epileptiform discharges reflect neuronal hyperexcitability, which may arise in or around ischemic tissue or in areas at risk of ischemia [25, 26]. It is therefore not surprising that EEG is considered a promising modality for monitoring and predicting DCI. EEG abnormalities generally appear early, preceding clinical DCI by hours to several days and often between days 3 and 7 [21, 22, 26, 37, 57, 59, 88, 93]. These temporal findings should, however, be interpreted in light of the heterogeneity in outcome definitions used across studies (Supplementary Table S5).

Among EEG studies, qEEG has been the most frequently studied method. It is particularly well-suited for prolonged recordings, as it enables automated processing and consistent interpretation of large datasets. While methodological details vary between studies, the alpha/delta ratio has shown the strongest predictive performance, with several studies reporting AUC values exceeding 0.80 [12, 14, 59]. One study employing predefined alarm criteria also used the alpha/delta ratio and reported a good predictive accuracy (sensitivity 0.96, specificity 0.80) [57]. In contrast, studies that focused solely on the presence of epileptiform discharges showed clearly inferior predictive performance [26].

In conclusion, qEEG is the most widely used EEG analysis technique in the context of scalp EEG for DCI prediction. The alpha/delta ratio appears to be the most promising marker, consistently demonstrating good predictive value across studies. Other qEEG parameters have been studied less extensively and generally show lower diagnostic performance. Ultimately, it would be logical to move from purely quantitative analysis to semiquantitative approaches based on validated cutoff thresholds to support real-time clinical decision-making.

## Discussion

### Summary of key findings

This scoping review identified 46 studies evaluating three neurophysiological modalities for the prediction and detection of DCI. No single modality demonstrated superiority above another; each modality provided complementary insights into the pathophysiology of DCI. TCD is the most extensively studied and commonly used modality, primarily assessing large artery hemodynamics. The MCA-MFV is most frequently used, though with variable thresholds and predictive performance. NIRS provides a distinct perspective by measuring cerebral oxygenation, reflecting microvascular and metabolic compromise. ROC-based rSO₂ cutoffs derived from study-specific data demonstrated the highest diagnostic accuracy. EEG, particularly qEEG, detects cortical hypoactivity and asymmetry, with the alpha/delta ratio consistently showing substantial predictive value for DCI.

### Comparison

Recent modality-specific meta-analyses are largely in line with our findings. TCD-derived biomarkers, particularly mean CBFV combined with the Lindegaard ratio and autoregulatory markers, appear promising for DCI prediction, although substantial heterogeneity and risk of bias remain, and pooled analyses may not fully capture the range of alternative TCD approaches used in clinical research [61]. NIRS-based measures likewise support a potential role in DCI detection, but diagnostic accuracy appears only moderate and is limited by heterogeneous thresholds and study methods [4]. Both TCD and EEG have also shown favorable pooled diagnostic performance, with EEG appearing more sensitive and TCD providing a more balanced sensitivity–specificity profile [11]. Our review complements these modality-specific meta-analyses by comparing modalities not only in terms of diagnostic performance, but also with respect to their physiological targets, analytic approaches, outcome definitions, and temporal aspects in relation to DCI.

### Comparison with radiological imaging

Although this scoping review was not designed to systematically evaluate head-to-head comparisons between diagnostic modalities, a few included studies incorporated such comparisons. One comparative study evaluating TCD and CTA found that CTA consistently demonstrated vasospasm, rendering it less predictive of DCI compared to TCD [76]. In contrast, another study comparing TCD with CTA, 4D-CTA, and CTP reported that TCD was less accurate in predicting DCI [44]. A third study comparing CTP and TCD found that CTP successfully predicted DCI, whereas TCD did not [49]. Given the limited number of comparative studies and the inconsistency of results, no definitive conclusions can be drawn regarding which modality offers superior predictive value for DCI.

### Temporal relationship between monitoring abnormalities and DCI onset

DCI typically develops within the first two weeks after the ictus, particularly at days 4–14 [16, 20, 31]. Ideally, a screening modality should predict an evolving risk of DCI early enough to allow targeted intervention. Importantly, clinical relevance depends on the reference outcome. Imaging-based infarction endpoints are often retrospective and less directly actionable than clinical DCI.

Although diagnostic performance is often reported, many studies do not provide an in-depth analysis of the temporal relationship between monitoring abnormalities and DCI onset. Clinically defined DCI can be identified during routine neurological examinations, enabling recognition close to onset. In contrast, DCI based on imaging is typically determined retrospectively, often weeks later. Consequently, the exact time of onset is uncertain**.** The same limitation applies to combined outcomes that include an imaging component. In patients with impaired consciousness, diagnosis of DCI relies primarily on imaging, which complicates determination of the interval between monitoring abnormalities and DCI onset. Accordingly, the interval between monitoring abnormalities and DCI onset should be interpreted in light of the reference outcome, as infarction-based endpoints are typically retrospective and may not capture the onset of evolving ischemia.

Since DCI is multifactorial, there is no a priori reason to favor one modality as the earliest indicator. Vascular, metabolic, and electrophysiologic processes may differ in timing, and measurable signals may vary in their temporal relation to DCI onset. Of these, the pre-onset interval between signal change and DCI onset is best characterized for EEG, with several studies suggesting abnormalities can precede clinical deterioration by hours to days. However, based on current evidence, it is not possible to determine which modality consistently alarms first. In studies that monitored EEG and TCD concurrently, earlier EEG changes have been observed. However, these comparisons are confounded by heterogeneity in TCD implementation, such as sampling frequency, vessel coverage, and whether optimized early-detection criteria were applied [21, 22].

### Strengths and limitations of the evidence

A key strength of this scoping review lies in its ability to accommodate heterogeneity across studies without the need for formal quality assessment. This flexibility enabled the inclusion of a wide range of analyses across modalities, capturing diverse phenomena and varying signal analysis techniques. As a result, this review provides a broad overview of what has been investigated within each modality, the analytical methods employed, and the diagnostic outcomes reported. This breadth of evidence highlights both the current state and the future potential of neurophysiological approaches to improve DCI diagnostics.

Several limitations should also be acknowledged. Because the methodological quality of the included studies was not formally appraised, caution is warranted when interpreting diagnostic accuracy metrics such as sensitivity, specificity, and AUC. We considered it informative to summarize these values and use them to guide interpretation, but definitive conclusions cannot be drawn. Although consensus definitions have been proposed, the literature still shows broad variability in how DCI and related outcomes are defined, and we therefore included studies across this spectrum [41, 60, 76, 80, 82]. Our inclusion criteria, which followed the STARD guidelines, further limited eligible studies [7]. Reports describing associations with DCI in terms of odds ratios rather than diagnostic accuracy metrics were excluded, although these may still hold clinical value. In addition, only 3 of the 46 identified diagnostic modalities were included, while more complex approaches such as machine learning–based prediction models were beyond the scope of this review. Nearly all included studies were single-center, most were retrospective, and some were based on small populations. External validation was lacking, and in certain cases the exact device used was not specified, limiting reproducibility and comparability. Finally, we selected methodologically representative studies to illustrate the range of diagnostic accuracy, but this pragmatic approach, rather than formal evidence grading, may have introduced some selection bias.

### Clinical implications

While each modality has inherent limitations, available evidence supports their potential for early risk stratification and underscores the value of neurophysiological monitoring in the neurocritical care of aSAH patients. Clinical interpretation should also take into account that reported diagnostic performance depends on the DCI-related outcome definition and on the timing of neurophysiological changes relative to DCI onset.

Across all modalities, ROC-based thresholds consistently perform well, and models that determine their own cutoff values also often yield better results. This is not unexpected, as such approaches enable appropriate calibration of the specific technique and analytical method to the study population, allowing for context-specific optimization. We therefore recommend that, when applying neurophysiological diagnostics, such calibration should be performed.

Based on this review, we are unable to indicate a specific preference for one modality when the choice is limited to a single option. TCD offers the advantage of already being one of the most widely used techniques for monitoring aSAH patients in the prevention of DCI. In general, TCD is readily accessible, cost-effective, feasible at the bedside, and allows for continuous monitoring. However, continuous TCD monitoring in awake patients presents certain limitations, particularly regarding patient burden and maintaining signal stability. As a result, intermittent snapshot measurements are often preferred in clinical practice. In addition to patient discomfort, TCD is also operator dependent.

NIRS has several advantages: it is well-suited for bedside application, minimally burdensome for the patient, largely independent of the operator, and generally well tolerated during continuous monitoring. Although NIRS offers several practical advantages, its implementation in hospital settings for adult neurological patients remains less widespread compared to TCD and EEG. A limitation, however, is that NIRS measurements for predicting DCI have thus far been restricted to relatively limited regions, primarily the frontal cortex. Expanding the monitored brain regions would be valuable for future studies.

EEG is a well-established technique with extensive clinical experience, including prolonged recordings, bedside, and ambulatory EEG monitoring. Hospitals providing care for neurological patients are generally equipped with this modality. The use of qEEG enables interpretation of EEG data by non-neurophysiologists, which may facilitate broader clinical implementation. EEG is generally well tolerated by patients and is not strongly operator-dependent; however, it is susceptible to artifacts, particularly those caused by patient movement and electrical interference from the surrounding environment.

Some studies suggest that combining diagnostic modalities may lead to improved predictive performance. For instance, one study that evaluated TCD and qEEG both independently and in combination showed that the alpha/delta ratio from qEEG alone yielded an AUC of 0.85 (95% CI 0.79–0.93), while TCD MCA-MFV alone had an AUC of 0.80 (95% CI 0.79–0.83). When combined, the AUC increased to 0.92 (95% CI 0.90–0.99), indicating that while TCD and qEEG alone did not differ significantly, their combination appeared to enhance predictive accuracy [14]. Similarly, a study comparing TCD alone to a combined approach with DSA found that the combination resulted in a slightly higher sensitivity of 0.93, although at the cost of a lower specificity of 0.68 compared to TCD alone [51]. Additional studies demonstrated the added value of combining TCD with other physiological parameters. For example, combining TCD with melatonin levels [67] or with arterial blood pressure [74] also showed potential for improving prediction.

In addition to risk prediction, neuromonitoring may also support therapeutic decision-making, for example, by tailoring blood pressure targets to cerebral perfusion or by triggering additional imaging or endovascular therapy when indicated. [5, 53, 64, 83, 90] As such, these techniques may contribute both to earlier identification of patients at risk and to more individualized strategies for initiating or withholding treatment. Nevertheless, current evidence that such approaches improve patient outcomes remains limited [55, 70, 83].

### Directions for future research

Future studies should aim to validate modality-specific cutoff values in larger, prospective, multicenter cohorts. It is important to recognize that some of these thresholds may be center-specific due to differences in measurement equipment and patient populations.

Standardized reporting of DCI definitions and outcome measures is still lacking and remains an essential unmet need in the field [82]. This lack of consistency hampers cross-study comparisons and limits the generalizability of findings. The temporal relationship between neurophysiological abnormalities and DCI-related outcomes should also be defined and reported more consistently, including the interval between signal changes and clinical or radiological endpoints. Studies evaluating the added value of multimodal monitoring, particularly through the integration of TCD, NIRS, and EEG, are important and should be conducted. Continuous monitoring is likely preferable to intermittent snapshot assessments, as it may offer more detailed and timely detection of evolving pathophysiological changes.

Advances in data analytics are increasingly relevant for neurophysiological monitoring. Artificial intelligence (AI) methods play a central role, particularly machine learning approaches such as deep learning. These techniques have the potential to enhance predictive performance and support real-time decision-making in neurocritical care settings [36, 46]. Beyond improving predictive accuracy, these approaches can facilitate automated interpretation of TCD, NIRS, and EEG signals, reduce interobserver variability, support artifact detection, and enable continuous real-time analysis at the bedside. Such developments could substantially increase the clinical utility and scalability of neurophysiological monitoring in patients with aSAH 48, 69, 71, 72]. [Recent advances in automated EEG interpretation further highlight this potential, including hybrid AI systems achieving expert-level accuracy in background analysis and report generation, [73] and AI models for real-time detection of cerebral ischemia in unconscious patients, which are currently under development [1, 6].

In addition to accuracy analyses for DCI prediction by individual modalities, future efforts should also focus on developing risk models that estimate the probability of DCI based on multiple input variables. Machine learning approaches are particularly suited for such tasks, as these models can operate in real time, continuously updating the predicted risk as new physiological data become available.

The ultimate aim of monitoring is not merely the prediction of DCI but its prevention, and future research should establish whether neuromonitoring-guided clinical decisions translate into improved patient outcomes.

## Conclusions

TCD, NIRS, and EEG contribute to the detection of DCI, and their combined use may enhance diagnostic accuracy. Each modality provides valuable and complementary insights into the pathophysiology of DCI in patients with aSAH. TCD remains the most established modality; however, variability in cutoff values hinders standardization. NIRS and EEG, though less routinely implemented, demonstrate promising diagnostic accuracy and offer distinct physiological perspectives, namely, cerebral oxygenation and neuronal activity, respectively. The temporal evolution of neurophysiological signals is relevant, as abnormalities in NIRS, TCD, and EEG may precede DCI by hours to days, providing a potential window for timely intervention. At the same time, diagnostic performance should be interpreted in the context of heterogeneous outcome definitions.

As no single modality has demonstrated universal superiority, future research should prioritize multimodal integration, the establishment of standardized thresholds, the role of AI in interpretation and the development of risk models that estimate the probability of DCI development.

Optimizing neurophysiological monitoring protocols may ultimately improve early detection of DCI risk, guide timely therapeutic decisions, either to initiate or withhold interventions, and enhance clinical outcomes in patients with aSAH if validated in prospective outcome studies.

## Supplementary Information

Below is the link to the electronic supplementary material.

ESM 1 Supplementary Material 1 (DOCX 56.5 KB)

## Data Availability

This scoping review used data extracted from previously published studies. Extracted data supporting the conclusions are provided in the article and supplementary materials. Additional extraction details are available from the corresponding author upon reasonable request.

## Abbreviations

ACA: Anterior cerebral artery
AI: Artificial intelligence
aSAH: Aneurysmal subarachnoid hemorrhage
AUC: Area under the receiver operating characteristic curve
BA: Basilar artery
BSI: Brain symmetry index
cEEG: Continuous electroencephalography
CSF: Cerebrospinal fluid
CT: Non-contrast computed tomography
CTA: Computed tomography angiography
CTP: Computed tomography perfusion
CW: Continuous-wave
CW-NIRS: Continuous-wave NIRS
DCI: Delayed cerebral ischemia
DSA: Digital subtraction angiography
EDV: End-diastolic velocity
EEG: Electroencephalography
FD-NIRS: Frequency-domain NIRS
FFT: Fast Fourier Transformation
HHb: Deoxyhemoglobin
ICA: Internal carotid artery
MAP: Mean arterial pressure
MCA: Middle cerebral artery
MFV: Mean flow velocity
MRI: Magnetic resonance imaging
MTT: Mean transit time
NIRS: Near-infrared spectroscopy
NPV: Negative predictive value
O₂Hb: Oxyhemoglobin
PCA: Posterior cerebral artery
PICOS: Population, intervention, comparison, outcomes, study design
PPV: Positive predictive value
PRISMA-ScR: Preferred reporting items for systematic reviews and meta-analyses extension for scoping reviews
PSV: Peak systolic velocity
qEEG: Quantitative electroencephalography
ROC: Receiver operating characteristic
rSO₂: Regional cerebral oxygen saturation
SAH: Subarachnoid hemorrhage
SPECT: Single-photon emission computed tomography
STARD: Standards for reporting diagnostic accuracy studies
TCCD: Transcranial color-coded duplex
TCD: Transcranial Doppler
TD-NIRS: Time-domain NIRS
TTP: Time to peak
VA: Vertebral artery
Xe-CT: Xenon-enhanced computed tomography
ΔoxCCO: Redox state of oxidized cytochrome c oxidase
τ: Cerebral arterial time constant

## References

[1] Akras Z, Jing J, Westover MB et al (2025) Using artificial intelligence to optimize anti-seizure treatment and EEG-guided decisions in severe brain injury. Neurotherapeutics 22(1):e00524. 10.1016/j.neurot.2025.e00524 39855915 10.1016/j.neurot.2025.e00524 PMC11840355

[2] Al-Jehani H, Angle M, Marcoux J et al (2018) Early abnormal transient hyperemic response test can predict delayed ischemic neurologic deficit in subarachnoid hemorrhage. Crit Ultrasound J 10:1. 10.1186/s13089-017-0079-7 29302799 10.1186/s13089-017-0079-7 PMC5754282

[3] Balança B, Dailler F, Boulogne S et al (2018) Diagnostic accuracy of quantitative EEG to detect delayed cerebral ischemia after subarachnoid hemorrhage: a preliminary study. Clin Neurophysiol 129:1926–1936. 10.1016/j.clinph.2018.06.013 30007892 10.1016/j.clinph.2018.06.013

[4] Bensaidane MR, Turgeon AF, Lauzier F et al (2025) Neuromonitoring with near-infrared spectroscopy (NIRS) in aneurysmal subarachnoid hemorrhage: a systematic review and meta-analysis. Crit Care 29(1):487. 10.1186/s13054-025-05701-3 41239395 10.1186/s13054-025-05701-3 PMC12619181

[5] Beqiri E, Tas J, Czosnyka M et al (2025) Does targeting CPP at CPPopt actually improve cerebrovascular reactivity? A secondary analysis of the COGiTATE randomized controlled trial. Neurocrit Care 42:937–944. 10.1007/s12028-024-02168-y 39623160 10.1007/s12028-024-02168-y PMC12137505

[6] Block L, El-Merhi A, Liljencrantz J et al (2020) Cerebral ischemia detection using artificial intelligence (CIDAI)-a study protocol. Acta Anaesthesiol Scand 64:1335–1342. 10.1111/aas.13657 32533722 10.1111/aas.13657

[7] Bossuyt PM, Reitsma JB, Bruns DE et al (2015) STARD 2015: an updated list of essential items for reporting diagnostic accuracy studies. BMJ 351:h5527. 10.1136/bmj.h5527 26511519 10.1136/bmj.h5527 PMC4623764

[8] Bramer WM, Giustini D, de Jonge GB et al (2016) De-duplication of database search results for systematic reviews in EndNote. J Med Libr Assoc 104:240–243. 10.3163/1536-5050.104.3.014 27366130 10.3163/1536-5050.104.3.014 PMC4915647

[9] Carrera E, Schmidt JM, Oddo M et al (2009) Transcranial Doppler for predicting delayed cerebral ischemia after subarachnoid hemorrhage. Neurosurgery 65(2):316–323. 10.1227/01.Neu.0000349209.69973.88 19625911 10.1227/01.NEU.0000349209.69973.88

[10] Chang JJ, Triano M, Corbin MJ et al (2020) Transcranial Doppler velocity and associations with delayed cerebral ischemia in aneurysmal subarachnoid hemorrhage. J Neurol Sci 415:116934. 10.1016/j.jns.2020.116934 32526525 10.1016/j.jns.2020.116934

[11] Chen W, Guo X, Zhang Y (2026) Diagnostic accuracy of transcranial Doppler and electroencephalogram for delayed cerebral ischemia following aneurysmal subarachnoid hemorrhage: a systematic review and meta-analysis. J Clin Ultrasound. 10.1002/jcu.70175 41502031 10.1002/jcu.70175

[12] Claassen J, Hirsch LJ, Kreiter KT et al (2004) Quantitative continuous EEG for detecting delayed cerebral ischemia in patients with poor-grade subarachnoid hemorrhage. Clin Neurophysiol 115:2699–2710. 10.1016/j.clinph.2004.06.017 15546778 10.1016/j.clinph.2004.06.017

[13] Dabecco R, Gigliotti MJ, Mao G et al (2021) Transcranial Dopplers revisited: development of novel markers for cerebral vasospasm after aneurysmal subarachnoid hemorrhage. Cureus 13:e13605. 10.7759/cureus.13605 33816004 10.7759/cureus.13605 PMC8011464

[14] Dai Z, Zhang L, Liu X et al (2024) Predictive value of quantitative electroencephalogram combined with transcranial Doppler ultrasound in delayed cerebral ischemia after subarachnoid hemorrhage. World Neurosurg 186:e48–e53. 10.1016/j.wneu.2024.01.150 38310949 10.1016/j.wneu.2024.01.150

[15] Dankbaar JW, Rijsdijk M, van der Schaaf IC et al (2009) Relationship between vasospasm, cerebral perfusion, and delayed cerebral ischemia after aneurysmal subarachnoid hemorrhage. Neuroradiology 51:813–819. 10.1007/s00234-009-0575-y 19623472 10.1007/s00234-009-0575-y PMC2773037

[16] Dodd WS, Laurent D, Dumont AS et al (2021) Pathophysiology of delayed cerebral ischemia after subarachnoid hemorrhage: a review. J Am Heart Assoc 10:e021845. 10.1161/JAHA.121.021845 34325514 10.1161/JAHA.121.021845 PMC8475656

[17] Etminan N, Chang HS, Hackenberg K et al (2019) Worldwide incidence of aneurysmal subarachnoid hemorrhage according to region, time period, blood pressure, and smoking prevalence in the population: a systematic review and meta-analysis. JAMA Neurol 76:588–597. 10.1001/jamaneurol.2019.0006 30659573 10.1001/jamaneurol.2019.0006 PMC6515606

[18] Ferrari M, Mottola L, Quaresima V (2004) Principles, techniques, and limitations of near infrared spectroscopy. Can J Appl Physiol 29:463–487. 10.1139/h04-031 15328595 10.1139/h04-031

[19] Fung C, Heiland DH, Reitmeir R et al (2022) Ultrasound perfusion imaging for the detection of cerebral hypoperfusion after aneurysmal subarachnoid hemorrhage. Neurocrit Care 37:149–159. 10.1007/s12028-022-01460-z 35211837 10.1007/s12028-022-01460-z PMC9283360

[20] Geraghty JR, Testai FD (2017) Delayed cerebral ischemia after subarachnoid hemorrhage: beyond vasospasm and towards a multifactorial pathophysiology. Curr Atheroscler Rep 19:50–x. 10.1007/s11883-017-0690-x 29063300 10.1007/s11883-017-0690-x

[21] Gollwitzer S, Müller TM, Hopfengärtner R et al (2019) Quantitative EEG after subarachnoid hemorrhage predicts long-term functional outcome. J Clin Neurophysiol 36:25–31. 10.1097/wnp.0000000000000537 30418267 10.1097/WNP.0000000000000537

[22] Gollwitzer S, Groemer T, Rampp S et al (2015) Early prediction of delayed cerebral ischemia in subarachnoid hemorrhage based on quantitative EEG: a prospective study in adults. Clin Neurophysiol 126:1514–1523. 10.1016/j.clinph.2014.10.215 25500193 10.1016/j.clinph.2014.10.215

[23] Han PY, Kim JH, Kang HI et al (2008) Is transcranial Doppler ultrasonography old-fashioned?: One institutional validity study. J Korean Neurosurg Soc 44:63–66. 10.3340/jkns.2008.44.2.63 19096694 10.3340/jkns.2008.44.2.63 PMC2588333

[24] Hoh BL, Ko NU, Amin-Hanjani S et al (2023) 2023 guideline for the management of patients with aneurysmal subarachnoid hemorrhage: a guideline from the American Heart Association/American Stroke Association. Stroke 20230522. 10.1161/STR.0000000000000436 10.1161/STR.0000000000000436 37212182

[25] Jordan KG (1999) Continuous EEG monitoring in the neuroscience intensive care unit and emergency department. J Clin Neurophysiol 16:14–39. 10.1097/00004691-199901000-00002 10082089 10.1097/00004691-199901000-00002

[26] Kim JA, Zheng WL, Elmer J et al (2022) High epileptiform discharge burden predicts delayed cerebral ischemia after subarachnoid hemorrhage. Clin Neurophysiol 141:139–146. 10.1016/j.clinph.2021.01.022 33812771 10.1016/j.clinph.2021.01.022 PMC8429508

[27] Kistka H, Dewan MC, Mocco J (2013) Evidence-based cerebral vasospasm surveillance. Neurol Res Int 2013:256713. 10.1155/2013/256713 23862061 10.1155/2013/256713 PMC3686086

[28] Lawson MF, Chi YY, Velat GJ et al (2010) Timing of aneurysm surgery: the International Cooperative Study revisited in the era of endovascular coiling. J Neurointerv Surg 2(2):131–134. 10.1136/jnis.2009.001172 21990592 10.1136/jnis.2009.001172

[29] Lee JY, Lee MS, Whang K et al (2006) Accuracy of transcranial Doppler sonography for predicting cerebral infarction in aneurysmal subarachnoid hemorrhage. J Clin Ultrasound 34:380–384. 10.1002/jcu.20269 16944480 10.1002/jcu.20269

[30] Liu GJ, Guo ZD, Sun XC et al (2018) Monitoring of the effect of cerebral autoregulation on delayed cerebral ischemia in patients with aneurysmal subarachnoid hemorrhage. World Neurosurg 118:E269–E275. 10.1016/j.wneu.2018.06.170 29969743 10.1016/j.wneu.2018.06.170

[31] Macdonald RL (2014) Delayed neurological deterioration after subarachnoid haemorrhage. Nat Rev Neurol 10(1):44–58. 10.1038/nrneurol.2013.246 24323051 10.1038/nrneurol.2013.246

[32] Malhotra K, Conners JJ, Lee VH et al (2014) Relative changes in transcranial Doppler velocities are inferior to absolute thresholds in prediction of symptomatic vasospasm after subarachnoid hemorrhage. J Stroke Cerebrovasc Dis 23:31–36. 10.1016/j.jstrokecerebrovasdis.2012.08.004 22959107 10.1016/j.jstrokecerebrovasdis.2012.08.004

[33] Milinis K, Thapar A, O’Neill K et al (2017) History of aneurysmal spontaneous subarachnoid hemorrhage. Stroke 48:e280–e283. 10.1161/STROKEAHA.117.017282 28808153 10.1161/STROKEAHA.117.017282

[34] Miller CM, Palestrant D, Schievink WI et al (2011) Prolonged transcranial Doppler monitoring after aneurysmal subarachnoid hemorrhage fails to adequately predict ischemic risk. Neurocrit Care 15:387–392. 10.1007/s12028-011-9564-1 21633870 10.1007/s12028-011-9564-1

[35] Moon H, Kwon J, Eun J et al (2024) Electrocorticogram (ECoG): engineering approaches and clinical challenges for translational medicine. Adv Mater Technol 9:2301692. 10.1002/admt.202301692

[36] Morid MA, Borjali A, Del Fiol G (2021) A scoping review of machine learning in health informatics and medical research. J Am Med Inform Assoc 28:576–584. 10.1093/jamia/ocaa321

[37] Mueller TM, Gollwitzer S, Hopfengärtner R et al (2021) Alpha power decrease in quantitative EEG detects development of cerebral infarction after subarachnoid hemorrhage early. Clin Neurophysiol 132:1283–1289. 10.1016/j.clinph.2021.03.005 33867261 10.1016/j.clinph.2021.03.005

[38] Murkin JM, Arango M (2009) Near-infrared spectroscopy as an index of brain and tissue oxygenation. Br J Anaesth 103(Suppl 1):3. 10.1093/bja/aep299 10.1093/bja/aep299 20007987

[39] Nakae R, Yokota H, Yoshida D et al (2011) Transcranial Doppler ultrasonography for diagnosis of cerebral vasospasm after aneurysmal subarachnoid hemorrhage: mean blood flow velocity ratio of the ipsilateral and contralateral middle cerebral arteries. Neurosurgery 69:876–883. 10.1227/NEU.0b013e318222dc4c 21558976 10.1227/NEU.0b013e318222dc4c

[40] Naqvi J, Yap KH, Ahmad G et al (2013) Transcranial Doppler ultrasound: a review of the physical principles and major applications in critical care. Int J Vasc Med 2013:629378. 10.1155/2013/629378 24455270 10.1155/2013/629378 PMC3876587

[41] Naraoka M, Matsuda N, Shimamura N et al (2022) Role of microcirculatory impairment in delayed cerebral ischemia and outcome after aneurysmal subarachnoid hemorrhage. J Cereb Blood Flow Metab 42:186–196. 10.1177/0271678X211045446 34496662 10.1177/0271678X211045446 PMC8721782

[42] Naval NS, Thomas CE, Urrutia VC (2005) Relative changes in flow velocities in vasospasm after subarachnoid hemorrhage: a transcranial Doppler study. Neurocrit Care 2:133–140. 10.1385/ncc:2:2:133 16159055 10.1385/NCC:2:2:133

[43] Nieuwkamp DJ, Setz LE, Algra A et al (2009) Changes in case fatality of aneurysmal subarachnoid haemorrhage over time, according to age, sex, and region: a meta-analysis. Lancet Neurol 8:635–642. 10.1016/S1474-4422(09)70126-7 19501022 10.1016/S1474-4422(09)70126-7

[44] Ognard J, Cheddad El Aouni M, Dissaux B et al (2020) Four-dimensional computed tomography angiography analysis of internal carotid arteries opacification at the skull base to detect delayed cerebral ischemia: a feasibility study. Int J Comput Assist Radiol Surg 15:2005–2015. 10.1007/s11548-020-02268-y 33026600 10.1007/s11548-020-02268-y

[45] Ouzzani M, Hammady H, Fedorowicz Z et al (2016) Rayyan-a web and mobile app for systematic reviews. Syst Rev 5(1):210. 10.1186/s13643-016-0384-4 27919275 10.1186/s13643-016-0384-4 PMC5139140

[46] Palmisciano P, Hoz SS, Johnson MD et al (2023) External validation of an extreme gradient boosting model for prediction of delayed cerebral ischemia after aneurysmal subarachnoid hemorrhage. World Neurosurg 175:e108–e114. 10.1016/j.wneu.2023.03.036 36914029 10.1016/j.wneu.2023.03.036

[47] Park JJ, Kim Y, Chai CL et al (2021) Application of near-infrared spectroscopy for the detection of delayed cerebral ischemia in poor-grade subarachnoid hemorrhage. Neurocrit Care 35:767–774. 10.1007/s12028-021-01223-2 33963480 10.1007/s12028-021-01223-2 PMC8104035

[48] Park JJ, Kim C, Jeon JP (2020) Monitoring of delayed cerebral ischemia in patients with subarachnoid hemorrhage via near-infrared spectroscopy. J Clin Med. 10.3390/jcm9051595 32456319 10.3390/jcm9051595 PMC7290832

[49] Pham M, Johnson A, Bartsch AJ et al (2007) CT perfusion predicts secondary cerebral infarction after aneurysmal subarachnoid hemorrhage. Neurology 69:762–765. 10.1212/01.wnl.0000267641.08958.1b 17709708 10.1212/01.wnl.0000267641.08958.1b

[50] Purkayastha S, Sorond F (2012) Transcranial Doppler ultrasound: technique and application. Semin Neurol 32:411–420. 10.1055/s-0032-1331812 23361485 10.1055/s-0032-1331812 PMC3902805

[51] Rabinstein AA, Friedman JA, Weigand SD et al (2004) Predictors of cerebral infarction in aneurysmal subarachnoid hemorrhage. Stroke 35:1862–1866. 10.1161/01.STR.0000133132.76983.8e 15218156 10.1161/01.STR.0000133132.76983.8e

[52] Rajajee V, Fletcher JJ, Pandey AS et al (2012) Low pulsatility index on transcranial Doppler predicts symptomatic large-vessel vasospasm after aneurysmal subarachnoid hemorrhage. Neurosurgery 70:1195–1206. 10.1227/NEU.0b013e3182417dca 22089755 10.1227/NEU.0b013e3182417dca

[53] Rass V, Helbok R (2021) How to diagnose delayed cerebral ischaemia and symptomatic vasospasm and prevent cerebral infarction in patients with subarachnoid haemorrhage. Curr Opin Crit Care 27:103–114. 10.1097/MCC.0000000000000798 33405414 10.1097/MCC.0000000000000798

[54] Rathakrishnan R, Gotman J, Dubeau F et al (2011) Using continuous electroencephalography in the management of delayed cerebral ischemia following subarachnoid hemorrhage. Neurocrit Care 14:152–161. 10.1007/s12028-010-9495-2 21207187 10.1007/s12028-010-9495-2

[55] Rodriguez EE, Zaccarelli M, Sterchele ED et al (2024) “NeuroVanguard”: a contemporary strategy in neuromonitoring for severe adult brain injury patients. Crit Care 28(1):104. 10.1186/s13054-024-04893-4 38561829 10.1186/s13054-024-04893-4 PMC10985991

[56] Romano JG, Rabinstein AA, Arheart KL et al (2008) Microemboli in aneurysmal subarachnoid hemorrhage. J Neuroimaging 18:396–401. 10.1111/j.1552-6569.2007.00215.x 18494776 10.1111/j.1552-6569.2007.00215.x

[57] Rosenthal ES, Biswal S, Zafar SF et al (2018) Continuous electroencephalography predicts delayed cerebral ischemia after subarachnoid hemorrhage: a prospective study of diagnostic accuracy. Ann Neurol 83:958–969. 10.1002/ana.25232 29659050 10.1002/ana.25232 PMC6021198

[58] Rossini PM, Cole J, Paulus W et al (2025) 1924-2024: first centennial of EEG. Clin Neurophysiol 170:132–135. 10.1016/j.clinph.2024.11.021 39718051 10.1016/j.clinph.2024.11.021

[59] Rots ML, van Putten MJ, Hoedemaekers CW et al (2016) Continuous EEG monitoring for early detection of delayed cerebral ischemia in subarachnoid hemorrhage: a pilot study. Neurocrit Care 24:207–216. 10.1007/s12028-015-0205-y 26432793 10.1007/s12028-015-0205-y

[60] Rynkowski CB, de Oliveira Manoel AL, Dos Reis MM et al (2019) Early Transcranial Doppler evaluation of cerebral autoregulation independently predicts functional outcome after aneurysmal subarachnoid hemorrhage. Neurocrit Care 31:253–262. 10.1007/s12028-019-00732-5 31102237 10.1007/s12028-019-00732-5

[61] Schenck H, van Craenenbroeck C, van Kuijk S et al (2025) Systematic review and meta-analysis of transcranial Doppler biomarkers for the prediction of delayed cerebral ischemia following subarachnoid hemorrhage. J Cereb Blood Flow Metab 45:1031–1047. 10.1177/0271678X251313746 40110695 10.1177/0271678X251313746 PMC11926817

[62] Scherle Matamoros CE, Samaniego EA, Sam K et al (2020) Prediction of Symptomatic Vasospasm in Patients with Aneurysmal Subarachnoid Hemorrhage Using Early Transcranial Doppler. J Vasc Interv Neurol 11:19–26 PMC6998809 32071668

[63] Scherschinski L, Catapano JS, Karahalios K et al (2022) Electroencephalography for detection of vasospasm and delayed cerebral ischemia in aneurysmal subarachnoid hemorrhage: a retrospective analysis and systematic review. Neurosurg Focus 52:E3. 10.3171/2021.12.FOCUS21656 35231893 10.3171/2021.12.FOCUS21656

[64] Silverman A, Kodali S, Strander S et al (2019) Deviation from personalized blood pressure targets is associated with worse outcome after subarachnoid hemorrhage. Stroke 50:2729–2737. 10.1161/STROKEAHA.119.026282 31495332 10.1161/STROKEAHA.119.026282 PMC6756936

[65] Sivakumar S, Tsetsou S, Patel AB et al (2022) Cortical spreading depolarizations and clinically measured scalp EEG activity after aneurysmal subarachnoid hemorrhage and traumatic brain injury. Neurocrit Care 37:49–59. 10.1007/s12028-021-01418-7 34997536 10.1007/s12028-021-01418-7 PMC9810077

[66] Snider SB, Migdady I, LaRose SL et al (2022) Transcranial-Doppler-measured vasospasm severity is associated with delayed cerebral infarction after subarachnoid hemorrhage. Neurocrit Care 36:815–821. 10.1007/s12028-021-01382-2 34751900 10.1007/s12028-021-01382-2

[67] Su Y, Cao Y, Zang H et al (2024) Combined transcranial Doppler and melatonin levels to predict delayed cerebral ischemia after subarachnoid hemorrhage. Neurologist 29:280–284. 10.1097/nrl.0000000000000565 38602912 10.1097/NRL.0000000000000565

[68] Suarez JI, Qureshi AI, Yahia AB et al (2002) Symptomatic vasospasm diagnosis after subarachnoid hemorrhage: evaluation of transcranial Doppler ultrasound and cerebral angiography as related to compromised vascular distribution. Crit Care Med 30:1348–1355. 10.1097/00003246-200206000-00035 12072693 10.1097/00003246-200206000-00035

[69] Toi H, Matsumoto N, Yokosuka K et al (2013) Prediction of cerebral vasospasm using early stage transcranial Doppler. Neurol Med Chir (Tokyo) 53:396–402. 10.2176/nmc.53.396 23803618 10.2176/nmc.53.396

[70] Treggiari MM, Rabinstein AA, Busl KM et al (2023) Guidelines for the neurocritical care management of aneurysmal subarachnoid hemorrhage. Neurocrit Care 39(1):1–28. 10.1007/s12028-023-01713-5 37202712 10.1007/s12028-023-01713-5

[71] Tribuddharat S, Ngamsaengsirisup K, Mahothorn P et al (2022) Correlation and agreement of regional cerebral oxygen saturation measured from sensor sites at frontal and temporal areas in adult patients undergoing cardiovascular anesthesia. PeerJ 10:e14058. 10.7717/peerj.14058 36128196 10.7717/peerj.14058 PMC9482766

[72] Tricco AC, Lillie E, Zarin W et al (2018) PRISMA extension for scoping reviews (PRISMA-ScR): checklist and explanation. Ann Intern Med 169:467–473. 10.7326/M18-0850 30178033 10.7326/M18-0850

[73] Tung CS, Liang SF, Chang SF et al (2025) A hybrid artificial intelligence system for automated EEG background analysis and report generation. IEEE J Biomed Health Inform 29(4):2629–2641. 10.1109/JBHI.2024.3496996 40030193 10.1109/JBHI.2024.3496996

[74] Uryga A, Kasprowicz M, Budohoski K et al (2024) Predictive value of cerebrovascular time constant for delayed cerebral ischemia after aneurysmal subarachnoid hemorrhage. J Cereb Blood Flow Metab 44:1208–1217. 10.1177/0271678x241228512 38295872 10.1177/0271678X241228512 PMC11179618

[75] van Donkelaar CE, Bakker NA, Veeger NJ et al (2015) Predictive factors for rebleeding after aneurysmal subarachnoid hemorrhage: Rebleeding Aneurysmal Subarachnoid Hemorrhage Study. Stroke 46:2100–2106. 10.1161/STROKEAHA.115.010037 26069261 10.1161/STROKEAHA.115.010037

[76] van der Harst JJ, Luijckx GR, Elting JWJ et al (2019) Transcranial Doppler Versus CT-Angiography for Detection of Cerebral Vasospasm in Relation to Delayed Cerebral Ischemia After Aneurysmal Subarachnoid Hemorrhage: A Prospective Single-Center Cohort Study: The Transcranial doppler and CT-angiography for Investigating Cerebral vasospasm in Subarachnoid hemorrhage (TACTICS) study. Crit Care Explor 1:e0001. 10.1097/cce.0000000000000001 32166226 10.1097/CCE.0000000000000001 PMC7063867

[77] van der Harst JJ, Elting JWJ, Hijlkema J et al (2024) Diagnostic value of transcranial Doppler to predict delayed cerebral ischemia after aneurysmal subarachnoid hemorrhage: to predict delayed cerebral ischemia. Acta Neurochir (Wien) 166:278. 10.1007/s00701-024-06164-1 38949680 10.1007/s00701-024-06164-1 PMC11217085

[78] van der Kleij LA, De Vis JB, Olivot JM et al (2017) Magnetic resonance imaging and cerebral ischemia after aneurysmal subarachnoid hemorrhage: a systematic review and meta-analysis. Stroke 48(1):239–245. 10.1161/STROKEAHA.116.011707 27924052 10.1161/STROKEAHA.116.011707

[79] van der Harst JJ, Elting JWJ, Bokkers RPH et al (2023) The diagnostic value of near-infrared spectroscopy to predict delayed cerebral ischemia and unfavorable outcome after subarachnoid hemorrhage. World Neurosurg 178:e202–e212. 10.1016/j.wneu.2023.07.033 37454906 10.1016/j.wneu.2023.07.033

[80] Veldeman M, Rossmann T, Haeren R et al (2024) Delayed cerebral infarction after aneurysmal subarachnoid hemorrhage: location, distribution patterns, infarct load, and effect on outcome. Neurology 103:e209607. 10.1212/WNL.0000000000209607 38950352 10.1212/WNL.0000000000209607

[81] Vergouwen MD, Vermeulen M, Coert BA et al (2008) Microthrombosis after aneurysmal subarachnoid hemorrhage: an additional explanation for delayed cerebral ischemia. J Cereb Blood Flow Metab 28:1761–1770. 10.1038/jcbfm.2008.74 18628782 10.1038/jcbfm.2008.74

[82] Vergouwen MD, Vermeulen M, van Gijn J et al (2010) Definition of delayed cerebral ischemia after aneurysmal subarachnoid hemorrhage as an outcome event in clinical trials and observational studies: proposal of a multidisciplinary research group. Stroke 41:2391–2395. 10.1161/STROKEAHA.110.589275 20798370 10.1161/STROKEAHA.110.589275

[83] Vitt JR, Loper NE, Mainali S (2023) Multimodal and autoregulation monitoring in the neurointensive care unit. Front Neurol 14:1155986. 10.3389/fneur.2023.1155986 37153655 10.3389/fneur.2023.1155986 PMC10157267

[84] Wang HC, Lin WC, Yang TM et al (2012) Time course of cerebral hemodynamics in aneurysmal subarachnoid hemorrhage. J Clin Ultrasound 40:91–98. 10.1002/jcu.20900 22102409 10.1002/jcu.20900

[85] Wang YL, Ma YQ, Hui PQ et al (2018) Evaluation of application value of Transcranial Doppler (TCD) in the inspection of cerebral vasospasm after the treatment of intracranial aneurysm. Curr Med Imaging Rev 14:143–146. 10.2174/1573405613666170504150537 29399014 10.2174/1573405613666170504150537 PMC5759172

[86] Washington CW, Zipfel GJ, Participants in the International Multi-disciplinary Consensus Conference on the Critical Care Management of Subarachnoid H (2011) Detection and monitoring of vasospasm and delayed cerebral ischemia: a review and assessment of the literature. Neurocrit Care 15:312–317. 10.1007/s12028-011-9594-8 21748499 10.1007/s12028-011-9594-8

[87] Westermaier T, Pham M, Stetter C et al (2014) Value of transcranial Doppler, perfusion-CT and neurological evaluation to forecast secondary ischemia after aneurysmal SAH. Neurocrit Care 20:406–412. 10.1007/s12028-013-9896-0 23982597 10.1007/s12028-013-9896-0

[88] Wickering E, Gaspard N, Zafar S et al (2016) Automation of classical QEEG trending methods for early detection of delayed cerebral ischemia: more work to do. J Clin Neurophysiol 33:227–234. 10.1097/wnp.0000000000000278 27258446 10.1097/WNP.0000000000000278 PMC4894333

[89] Wolf M, Ferrari M, Quaresima V (2007) Progress of near-infrared spectroscopy and topography for brain and muscle clinical applications. J Biomed Opt 12:062104. 10.1117/1.2804899 18163807 10.1117/1.2804899

[90] Xie J, Carbonara AR, Al-Battashi AW et al (2025) Individualized mean arterial pressure targets in critically ill patients guided by non-invasive cerebral-autoregulation: a scoping review. Crit Care 29(1):196. 10.1186/s13054-025-05432-5 40380314 10.1186/s13054-025-05432-5 PMC12084981

[91] Yang SY, Tan BB, Lin J et al (2024) Monitoring of perioperative microcirculation dysfunction by near-infrared spectroscopy for neurological deterioration and prognosis of aneurysmal subarachnoid hemorrhage: an observational, longitudinal cohort study. Neurol Ther 13:475–495 38367176 10.1007/s40120-024-00585-x PMC10951157

[92] Yousef KM, Balzer JR, Crago EA et al (2014) Transcranial regional cerebral oxygen desaturation predicts delayed cerebral ischaemia and poor outcomes after subarachnoid haemorrhage: a correlational study. Intensive Crit Care Nurs 30:346–352. 10.1016/j.iccn.2014.05.001 24933608 10.1016/j.iccn.2014.05.001 PMC4254376

[93] Zheng WL, Kim JA, Elmer J et al (2022) Automated EEG-based prediction of delayed cerebral ischemia after subarachnoid hemorrhage. Clin Neurophysiol 143:97–106. 10.1016/j.clinph.2022.08.023 36182752 10.1016/j.clinph.2022.08.023 PMC9847346

