# Supplementary material for: Noninvasive neurophysiological diagnostics of delayed cerebral ischemia in aneurysmal subarachnoid hemorrhage: a scoping review
Source: Acta Neurochir (Wien). 2026 May 11;168(1):151. doi: 10.1007/s00701-026-06901-8 (PMC13333557; doi:10.1007/s00701-026-06901-8)
Supplement: Supplementary file 1 — Supplementary Material 1 (DOCX 56.5 KB) [file 701_2026_6901_MOESM1_ESM.docx]

Supplement

Table of contents

[Methods S1. Search strategies 2](#_Toc224558997)

[Table S2. Diagnostic modalities identified in the literature for predicting delayed cerebral ischemia in aneurysmal subarachnoid hemorrhage 4](#_Toc224558998)

[Table S3. Number of articles published per year on diagnostic modalities for predicting Delayed Cerebral Ischemia 5](#_Toc224558999)

[Table S4. Number of articles retrieved per journal on modalities used to predict delayed cerebral ischemia in aneurysmal subarachnoid hemorrhage 6](#_Toc224559000)

[Table S5. Descriptives of included studies 9](#_Toc224559001)

[Table S6. Distribution of DCI-related outcome categories across included studies by monitoring modality 12](#_Toc224559002)

[Table S7. Included articles on transcranial doppler 12](#_Toc224559003)

[Table S8. Included articles on near-infrared spectroscopy 12](#_Toc224559004)

[Table S9. Included articles on electroencephalography 12](#_Toc224559005)

### Methods S1. Search strategies

**PubMed:**

("Subarachnoid Hemorrhage"[Mesh] OR (subarachnoid*[tiab] AND (hemorrhage*[tiab] OR haemorrhage*[tiab] OR bleeding[tiab])))

AND

("Diagnostic Imaging"[Mesh] OR "Electroencephalography"[Mesh] OR "diagnostic imaging" [sh] OR imaging[tiab] OR mri[tiab] OR “magnetic resonance” [tiab] OR ct[tiab] OR “computed tomograph*”[tiab] OR spectroscop*[tiab] OR NIRS[tiab] OR angiograph*[tiab] OR doppler[tiab] OR duplex[tiab] OR electroencephal*[tiab] OR eeg[tiab] OR ceeg[tiab] OR xray*[tiab] OR x-ray*[tiab] OR ultraso*[tiab] OR radiogra*[tiab] OR modalit*[tiab] OR multimodal*[tiab] OR monitor*[tiab] OR neuromonitor*[tiab] OR surveill*[tiab])

AND

("Brain Ischemia"[Mesh] OR "Vasospasm, Intracranial"[Mesh] OR dci[tiab] OR vasospasm*[tiab] OR ((brain*[tiab] OR intracranial[tiab] OR transcranial[tiab] OR cerebr*[tiab] OR neurol*[tiab] OR delayed[tiab]) AND (ischem*[tiab] OR ischaem*[tiab] OR infarction[tiab])))

AND

("Sensitivity and Specificity"[Mesh] OR  (sensitiv*[tiab] AND specific*[tiab]) OR ((diagnos*[tiab] OR detect*[tiab]) AND ("Reproducibility of Results"[Mesh:NoExp] OR sensitivity[tiab] OR reliab*[tiab] OR reproducib*[tiab] OR accura*[tiab])) OR predict*[tiab] OR "diagnostic value"[tiab] OR “roc curve”[tiab] OR “receiver operat*”[tiab] OR (area[tiab] AND under[tiab] AND curve[tiab]) OR “odds ratio”[tiab] OR “concordance”[tiab] OR “Likehood ratio”[tiab] OR “likelihood ratio”[tiab] OR “false positive”[tiab] OR “false negative”[tiab] OR correlat*[ti] OR associat*[ti] OR course[ti])

NOT

("Animals"[Mesh] NOT "Humans"[Mesh])

**Embase:**

('subarachnoid hemorrhage'/exp/mj OR (subarachnoid* AND (hemorrhage* OR haemorrhage* OR bleeding)):ab,ti,kw)

AND

('radiodiagnosis'/exp OR 'electroencephalogram'/exp OR 'echography'/exp OR 'nuclear magnetic resonance'/exp OR 'radiography'/exp OR 'echography'/exp OR (imaging OR mri OR ‘magnetic resonance’ OR ct OR ‘computed tomograph*’ OR spectroscop* OR NIRS OR angiograph* OR doppler OR duplex OR electroencephal* OR eeg OR ceeg OR xray* OR ‘x-ray*’ OR ultraso* OR radiogra* OR modalit* OR multimodal* OR monitor* OR neuromonitor* OR surveill*):ab,ti,kw)

AND

('brain ischemia'/exp/mj OR 'brain infarction'/exp/mj OR (dci OR vasospasm* OR ((brain OR intracranial OR transcranial OR cerebr* OR neurol* OR delayed) AND (ischem* OR ischaem* OR infarction))):ab,ti,kw)

AND

('sensitivity and specificity'/exp OR 'predictive value'/exp OR 'predictive validity'/exp OR 'receiver operating characteristic'/exp OR ((sensitiv* AND specific*) OR ((diagnos* OR detect*) AND (sensitivity OR reliab* OR reproducib* OR accura*)) OR predict* OR ‘diagnostic value’ OR ‘roc curve’ OR ‘receiver operat*’ OR (area AND under AND curve) OR ‘odds ratio’ OR ‘concordance’ OR ‘Likehood ratio’ OR ‘likelihood ratio’ OR ‘false positive’ OR ‘false negative’):ab,ti,kw OR (correlat* OR associat* OR course):ti)

NOT

(('animal'/exp NOT 'human'/exp) OR 'conference abstract'/it OR 'case report'/exp)

**Web of Science:**

TS=("subarachnoid*" AND ("hemorrhage* "OR "haemorrhage*" OR "bleeding"))

AND

TS=("imaging" OR "mri" OR “magnetic resonance” OR "ct" OR “computed tomograph*” OR "spectroscop*" OR "NIRS" OR "angiograph*" OR "doppler" OR "duplex" OR "electroencephal*" OR "eeg" OR "ceeg" OR "xray*" OR "x-ray*" OR "ultraso*" OR "radiogra*" OR "modalit*" OR "multimodal*" OR "monitor*" OR "neuromonitor*" OR "surveill*")

AND

TS=( "dci" OR "vasospasm*" OR (("brain" OR "intracranial" OR "cerebr*" OR "neurol*" OR "delayed" OR transcranial) AND ("ischem*" OR "ischaem*" OR "infarction*")))

AND

(TS=(("sensitiv*" AND "specific*") OR (("diagnos*" OR "detect*") AND ("sensitivity" OR "reliab*" OR "reproducib*" OR "accura*")) OR "predict*" OR "diagnostic value" OR “roc curve” OR “receiver operat*” OR ("area" AND "under" AND "curve") OR “odds ratio” OR “concordance” OR “Likehood ratio” OR “likelihood ratio” OR “false positive” OR “false negative”) OR TI=( "correlat*" OR "associat*" OR "course"))

AND

DT=( "Article")

NOT

TI=("animal" OR "animals" OR "rat" OR "rats" OR "mouse" OR "mice" OR "murine" OR "rodent*" OR "dog" OR "dogs" OR "canine" OR "pig" OR "pigs")

### Table S2. Diagnostic modalities identified in the literature for predicting delayed cerebral ischemia in aneurysmal subarachnoid hemorrhage

| Modality | Full text | Included |
| --- | --- | --- |
| Biomarkers | 218 |  |
| Computed Tomography (CT) | 96 |  |
| Transcranial Doppler (TCD) | 75 | 26 |
| CT Perfusion (CTP) | 63 |  |
| Digital Subtraction Angiography (DSA) | 27 |  |
| Electroencephalography (EEG) | 27 | 14 |
| Microdialysis | 19 |  |
| Multi Modal | 17 |  |
| Cerebral Autoregulation | 15 |  |
| Magnetic Resonance Imaging (MRI) | 14 |  |
| CT Angiography (CTA) | 11 |  |
| Single Photon Emission Computed Tomography (SPECT) | 8 |  |
| Machine Learning | 9 |  |
| Near-Infrared Spectroscopy (NIRS) | 9 | 6 |
| Xe-CT | 7 |  |
| Cardiac and central vascular functional alterations | 3 |  |
| Cerebral perfusion pressure (CPP) | 3 |  |
| Cortical thermal clearance | 3 |  |
| Electrocardiographic (ECG) changes after SAH | 3 |  |
| 99mTc-ECD | 2 |  |
| Arterial blood pressure increase | 3 |  |
| Cerbrovascular reactivity (CVR) | 2 |  |
| CFI and ELWI by transpulmonary thermodilution | 2 |  |
| flat-detector computed tomography (IV FDCT) angiography | 2 |  |
| Large negative direct current (DC)-electrocorticography (ECoG) | 2 |  |
| noninfectious fever | 2 |  |
| Positron Emission Tomography (PET) | 2 |  |
| Intracranial Pressure (ICP) and Cerebral Perfusion Pressure (CPP) | 1 |  |
| Ankle-Brachial Index | 1 |  |
| Aterial Blood gas analyses (paO2) | 1 |  |
| Arterial stiffness index | 1 |  |
| Arteriovenous Difference in Oxygen (AvDO2) | 1 |  |
| BIS monitoring | 1 |  |
| Blood viscosity | 1 |  |
| Global end-diastolic volume index | 1 |  |
| Index of the occurrence of atherosclerosis | 1 |  |
| Intraoperative measurement of cerebral blood flow | 1 |  |
| Jugular blood oxygen and lactate indices | 1 |  |
| Left ventricular dysfunction | 1 |  |
| Nimodipine-Induced Blood Pressure Changes | 1 |  |
| objective pupillometry | 2 |  |
| physiological time series data | 1 |  |
| retinal vessel analysis (RVA) | 1 |  |
| Saccadometry | 1 |  |
| somatosensory evoked potential (SEP) | 1 |  |
| Spontaneous Hyperventilation | 1 |  |
| Exlcuded (other) | 48 |  |
| Excluded (no modality prediction models, 22) | 25 |  |

### Table S3. Number of articles published per year on diagnostic modalities for predicting Delayed Cerebral Ischemia

| Year | Number of Articles |
| --- | --- |
| 2025 | 3 |
| 2024 | 196 |
| 2023 | 156 |
| 2022 | 208 |
| 2021 | 155 |
| 2020 | 168 |
| 2019 | 176 |
| 2018 | 175 |
| 2017 | 146 |
| 2016 | 129 |
| 2015 | 124 |
| 2014 | 117 |
| 2013 | 123 |
| 2012 | 113 |
| 2011 | 112 |
| 2010 | 98 |
| 2009 | 59 |
| 2008 | 83 |
| 2007 | 80 |
| 2006 | 73 |
| 2005 | 53 |
| 2004 | 55 |
| 2003 | 58 |
| 2002 | 52 |
| 2001 | 60 |
| 2000 | 33 |
| 1999 | 31 |
| 1998 | 34 |
| 1997 | 32 |
| 1996 | 37 |
| 1995 | 30 |
| 1994 | 27 |
| 1993 | 29 |
| 1992 | 16 |
| 1991 | 21 |
| 1990 | 15 |
| 1989 | 11 |
| 1988 | 5 |
| 1987 | 14 |
| 1986 | 12 |
| 1985 | 6 |
| 1984 | 13 |
| 1983 | 5 |
| 1982 | 7 |
| 1981 | 4 |
| 1980 | 10 |
| 1979 | 7 |
| 1978 | 6 |
| 1977 | 7 |
| 1976 | 2 |
| 1975 | 2 |
| 1974 | 3 |
| 1973 | 1 |
| 1969 | 1 |
| 1968 | 2 |
| 1966 | 1 |
| 1964 | 1 |

### Table S4. Number of articles retrieved per journal on modalities used to predict delayed cerebral ischemia in aneurysmal subarachnoid hemorrhage

| Journal | Number |
| --- | --- |
| Neurosurgery | 175 |
| Stroke | 165 |
| J Neurosurg | 163 |
| Neurocrit Care | 127 |
| World Neurosurg | 115 |
| J Stroke Cerebrovasc Dis | 75 |
| Acta Neurochir (Wien) | 54 |
| Neurocritical Care | 52 |
| Neurology | 48 |
| AJNR Am J Neuroradiol | 47 |
| Journal of Neurosurgery | 44 |
| World Neurosurgery | 41 |
| Acta Neurochir Suppl | 39 |
| Front Neurol | 38 |
| Neuroradiology | 35 |
| Neurosurg Rev | 33 |
| J Clin Neurosci | 30 |
| Neurol Med Chir (Tokyo) | 27 |
| Acta Neurochirurgica | 27 |
| Cerebrovasc Dis | 26 |
| J Neurol Neurosurg Psychiatry | 24 |
| Neurol Res | 23 |
| J Neurointerv Surg | 23 |
| Frontiers in Neurology | 22 |
| No Shinkei Geka | 22 |
| Clin Neurol Neurosurg | 21 |
| Surg Neurol | 20 |
| J Cereb Blood Flow Metab | 19 |
| Crit Care Med | 17 |
| J Neuroimaging | 16 |
| J Neurol Sci | 16 |
| J Clin Med | 16 |
| Neurochirurgie | 14 |
| Neurosurgical Review | 14 |
| Neurologia Medico-Chirurgica | 13 |
| Transl Stroke Res | 13 |
| Journal of Clinical Neuroscience | 13 |
| BMC Neurol | 12 |
| Sci Rep | 12 |
| Surg Neurol Int | 12 |
| Acta Neurochir. Suppl. | 12 |
| J Neuroradiol | 12 |
| American Journal of Neuroradiology | 12 |
| Crit Care | 11 |
| PLoS One | 11 |
| J Neurosurg Anesthesiol | 11 |
| Neurosurg Focus | 10 |
| Eur J Neurol | 10 |
| Cureus | 10 |
| Clin Neurophysiol | 10 |
| Journal of Cerebral Blood Flow and Metabolism | 9 |
| Clin Neuroradiol | 9 |
| J Neurol | 9 |
| Radiology | 9 |
| Critical Care | 9 |
| European Journal of Neurology | 8 |
| Eur J Radiol | 8 |
| Journal of the Neurological Sciences | 8 |
| J Clin Neurophysiol | 8 |
| Critical Care Medicine | 8 |
| Br J Neurosurg | 8 |
| Interv Neuroradiol | 8 |
| Cerebrovascular Diseases | 8 |
| Ann Neurol | 8 |
| Journal of Neurology Neurosurgery and Psychiatry | 8 |
| Surgical Neurology | 8 |
| Eur Radiol | 8 |
| Neurol India | 7 |
| No To Shinkei | 7 |
| Neurological Research | 7 |
| J Neurosurg Sci | 7 |
| Nervenarzt | 7 |
| Brain | 7 |
| Arq Neuropsiquiatr | 7 |
| Stroke-Vascular and Interventional Neurology | 6 |
| J Clin Monit Comput | 6 |
| Scientific Reports | 6 |
| Annals of Neurology | 6 |
| Journal of Korean Neurosurgical Society | 6 |
| Journal of Stroke and Cerebrovascular Diseases | 6 |
| International Journal of Clinical and Experimental Medicine | 6 |
| British Journal of Neurosurgery | 6 |
| Neurosurg Clin N Am | 6 |
| Can J Neurol Sci | 6 |
| Rev Neurol | 6 |
| Acta Neurol Scand | 5 |
| Journal of Neurointerventional Surgery | 5 |
| Clinical Neurology and Neurosurgery | 5 |
| Br J Anaesth | 5 |
| Am J Emerg Med | 5 |
| Clinical Neurophysiology | 5 |
| Canadian Journal of Neurological Sciences | 5 |
| Neuroradiol J | 5 |
| Bmc Neurology | 5 |
| Intensive Care Med | 5 |
| Ceska a Slovenska Neurologie a Neurochirurgie | 5 |
| Journal of Stroke & Cerebrovascular Diseases | 5 |
| medRxiv | 5 |
| Neurol Sci | 4 |
| Neurologia | 4 |
| Curr Neurovasc Res | 4 |
| Neurosurgery Quarterly | 4 |
| J Korean Neurosurg Soc | 4 |
| World Neurosurg X | 3 |
| Brain Hemorrhages | 3 |
| Brain Sci | 3 |
| Brain Behav | 2 |
| Brain Communications | 2 |
| Eur Stroke J | 2 |
| Heliyon | 2 |
| Neurol Med -Chir | 2 |
| Neurology and Therapy | 2 |
| Medicina (Kaunas) | 2 |
| CNS Neurosci Ther | 2 |
| Int J Stroke | 2 |
| AJR Am J Roentgenol | 2 |
| Acta Neurochir | 2 |
| Quant Imaging Med Surg | 2 |
| World J Radiol | 2 |
| Neuroradiology Journal | 2 |
| Neurocirugia | 2 |
| Eur Neurol | 1 |
| Operative Neurosurgery | 1 |
| International Journal of Endocrinology | 1 |
| Neuroimage | 1 |
| Neurological Sciences | 1 |
| Ultraschall in Der Medizin | 1 |
| Biomed Rep | 1 |
| Canadian Association of Radiologists Journal-Journal De L Association Canadienne Des Radiologistes | 1 |
| Am J Neuroradiol | 1 |
| Journal of Thoracic Disease | 1 |
| J Craniofac Surg | 1 |
| J Inflamm Res | 1 |
| Health Sci Rep | 1 |
| Eur Radiol Exp | 1 |
| Diagnostics (Basel) | 1 |
| Pol J Radiol | 1 |
| Semin Neurol | 1 |
| Arterioscler Thromb Vasc Biol | 1 |
| Inflammation | 1 |
| J Intensive Care Med | 1 |
| Interventional Neurology | 1 |
| Hong Kong Journal of Radiology | 1 |
| Rev Clin Esp (Barc) | 1 |
| Nihon Hoshasen Gijutsu Gakkai Zasshi | 1 |
| International Journal of General Medicine | 1 |
| J Obstet Gynaecol India | 1 |
| Crit Care Explor | 1 |
| Neuro-Oncol Pract | 1 |
| Asian J Pharm Clin Res | 1 |
| Brain Circ | 1 |
| PLoS ONE | 1 |
| Ann Afr Med | 1 |
| Front Surg | 1 |
| Aktuelle Kardiologie | 1 |
| J Am Heart Assoc | 1 |

### Table S5. Descriptives of included studies

| Author | Year | Study type | Modality | N | Definition of DCI | Outcome categories* | Number of DCI patients (% of total) |
| --- | --- | --- | --- | --- | --- | --- | --- |
| Carrera | 2009 | Retrospective | TCD | 441 | New focal deficit or CT infarction attributed to vasospasm by consensus review | III Radiological and/or clinical criteria | 92 (21%) |
| Chang | 2020 | Retrospective | TCD | 95 | Focal infarction on CT or MRI corresponding to vascular territories/watershed zones at discharge | I Delayed cerebral infarction on imaging | 24 (25%) |
| Dabecco | 2021 | Retrospective | TCD | 103 | Neurologic deterioration not explained by other causes | IV Clinical deterioration attributed to DCI | 13 (13%) |
| Dai | 2024 | Prospective | TCD | 105 | New focal neurologic deficits with MCA flow >120 cm/s on TCD and CT/CT perfusion showing infarction/ischemia | V Clinical symptoms and vasospasm | 34 (32%) |
| Fung | 2022 | Prospective | TCD | 30 | New focal deficits or ≥2-point GCS drop for ≥1 hour, excluding other causes | IV Clinical deterioration attributed to DCI | 12 (40%) |
| Han | 2008 | Prospective | TCD | 40 | Clinical deterioration (confusion, disorientation, focal deficits) after day 3 post-SAH, excluding other causes | IV Clinical deterioration attributed to DCI | 12 (30%) |
| Lee | 2006 | Retrospective | TCD | 93 | New hypodensity on CT in a vascular territory with corresponding clinical symptoms and exclusion of other causes | II Delayed cerebral infarction with clinical symptoms; | 23 (25%) |
| Malhotra | 2014 | Retrospective | TCD | 211 | New focal neurologic signs or consciousness deterioration with MCA vasospasm on angiography and endovascular treatment | V Clinical symptoms and vasospasm | 48 (23%) |
| Miller | 2011 | Retrospective | TCD | 107 | New hypodensity on CT corresponding to vascular territory and not present on admission or early follow-up scan | I Delayed cerebral infarction on imaging | 31 (29%) |
| Nakae | 2011 | Retrospective | TCD | 142 | New CT hypodensity in vascular territory or symptoms due to vasospasm (other causes excluded) | III Radiological and/or clinical criteria | 28 (20%) |
| Naval | 2005 | Retrospective | TCD | 50 | New focal signs or mental status change with moderate/severe vasospasm on angiography; other causes excluded | V Clinical symptoms and vasospasm | 10 (20%) |
| Ognard | 2020 | Retrospective | TCD | 10 | Delayed neurologic deterioration or infarction on imaging not explained by other causes | III Radiological and/or clinical criteria | 5 (50%) |
| Pham | 2007 | Prospective | TCD | 38 | New infarction on CT between days 3–14 post-SAH | I Delayed cerebral infarction on imaging | 14 (37%) |
| Rabinstein | 2004 | Retrospective | TCD | 143 | New hypodensity in vascular distribution on CT | I Delayed cerebral infarction on imaging | 57 (40%) |
| Rajajee | 2012 | Retrospective | TCD | 81 | Acute neuro deterioration + angiographic vasospasm | V Clinical symptoms and vasospasm | 21 (26%) |
| Romano | 2008 | Prospective | TCD | 40 | Neurological deterioration not due to other causes | IV Clinical deterioration attributed to DCI | 19 (48%) |
| Scherle Matamoros | 2020 | Retrospective | TCD | 51 | Temporary or permanent neurological deficit due to vasospasm | IV Clinical deterioration attributed to DCI | 12 (24%) |
| Snider | 2022 | Retrospective | TCD | 262 | New infarction >48h post-procedure not otherwise explained | I Delayed cerebral infarction on imaging | 27 (10%) |
| Su | 2024 | Prospective | TCD | 120 | New focal neurologic deficits with MCA flow >120 cm/s on TCD and CT/CT perfusion showing infarction/ischemia | V Clinical symptoms and vasospasm | 40 (33%) |
| Toi | 2013 | Prospective | TCD | 45 | New focal neurological deficits, LOC or both attributed to vasospasm | IV Clinical deterioration attributed to DCI | 11 (24%) |
| Uryga | 2024 | Retrospective | TCD | 71 | Focal neurological deficit or infarction attributable to secondary ischemia | III Radiological and/or clinical criteria | 17 (24%) |
| van der Harst | 2024 | Retrospective | TCD | 621 | New focal deficits, reduced LOC, or new infarction excluding other causes | III Radiological and/or clinical criteria | 55 (9%) |
| van der Harst | 2019 | Prospective | TCD | 59 | New focal deficits or ischemic lesions not otherwise explained | III Radiological and/or clinical criteria | 16 (27%) |
| Wang | 2012 | Prospective | TCD | 18 | New infarction on MRI | I Delayed cerebral infarction on imaging | 9 (50%) |
| Wang Y | 2018 | Retrospective | TCD | 105 | Neurological deterioration not due to other causes | IV Clinical deterioration attributed to DCI | 50 (48%) |
| Westermaier | 2014 | Prospective | TCD | 61 | New infarction on CT or angiographic vasospasm | V Clinical symptoms and vasospasm | 10 (16%) |
| Liu | 2018 | Prospective | NIRS | 81 | Neurological deterioration not attributable to other causes + imaging evidence | IV Clinical deterioration attributed to DCI | 45 (56%) |
| Park | 2020 | Prospective | NIRS | 52 | New neurological deficits + severe angiographic cerebral vasospasm | V Clinical symptoms and vasospasm | 18 (35%) |
| Park | 2021 | Prospective | NIRS | 24 | Neurological deterioration + severe vasospasm on CTA/MRA | V Clinical symptoms and vasospasm | 8 (33%) |
| van der Harst | 2023 | Retrospective | NIRS | 41 | New focal deficit or infarction on imaging excluding other causes | III Radiological and/or clinical criteria | 12 (29%) |
| Yang | 2024 | Retrospective | NIRS | 252 | Early/delayed neurological deterioration (NIHSS ≥4) | IV Clinical deterioration attributed to DCI | 94 (37%) |
| Yousef | 2014 | Prospective | NIRS | 163 | Neurological deterioration + impaired CBF (TCD, CT/CTP, angiography) | V Clinical symptoms and vasospasm | 93 (57%) |
| Balança | 2018 | Retrospective | EEG | 15 | New infarction on follow-up imaging not present 24–48h after occlusion | I Delayed cerebral infarction on imaging | 9 (60%) |
| Claassen | 2004 | Prospective | EEG | 34 | New clinical deterioration or infarct on CT not explained by other causes | III Radiological and/or clinical criteria | 9 (26%) |
| Dai | 2024 | Retrospective | EEG | 105 | New focal neurologic deficits with MCA flow >120 cm/s on TCD and CT/CT perfusion showing infarction/ischemia | V Clinical symptoms and vasospasm | 34 (32%) |
| Gollwitzer | 2019 | Prospective | EEG | 22 | New ischemic lesions on imaging (radiologist-assessed), not present in early imaging | I Delayed cerebral infarction on imaging | 16 (73%) |
| Gollwitzer | 2015 | Prospective | EEG | 12 | New ischemic lesion on imaging, not present on early imaging | I Delayed cerebral infarction on imaging | 6 (50%) |
| Kim | 2022 | Retrospective | EEG | 113 | Consensus definition: clinical deterioration or new infarct not due to other causes | III Radiological and/or clinical criteria | 58 (51%) |
| Mueller | 2021 | Retrospective | EEG | 34 | New ischemic lesion on CT or MRI within six weeks not present within 48h after occlusion | I Delayed cerebral infarction on imaging | 9 (26%) |
| Rathakrishnan | 2011 | Prospective | EEG | 12 | New unexplained focal deficit and/or silent infarction on CT | I Delayed cerebral infarction on imaging | 8 (67%) |
| Rosenthal | 2018 | Prospective | EEG | 103 | Consensus adjudicated DCI (clinical deterioration or infarct) | III Radiological and/or clinical criteria | 52 (50%) |
| Rots | 2016 | Prospective | EEG | 20 | Clinical deterioration or new infarct on CT | III Radiological and/or clinical criteria | 11 (55%) |
| Scherschinski | 2022 | Retrospective | EEG | 77 | New deficit or infarct after excluding other causes | III Radiological and/or clinical criteria | 71 (92%) |
| Sivakumar | 2022 | Prospective | EEG | 20 | New focal deficit or infarct not present in early imaging and not due to other causes | III Radiological and/or clinical criteria | 11 (55%) |
| Wickering | 2016 | Retrospective | EEG | 95 | Combined clinical deterioration and radiologic infarction | III Radiological and/or clinical criteria | 43 (45%) |
| Zheng | 2022 | Retrospective | EEG | 113 | Consensus: new deficit or infarct not explained by other causes | III Radiological and/or clinical criteria | 58 (51%) |
| * Across all categories, definitions required exclusion of alternative causes for the observed clinical and/or radiological findings. | | | | | | | |

### Table S6. Distribution of DCI-related outcome categories across included studies by monitoring modality

|  | I.  DCI on imaging | II.  DCI with clinical symptoms) | III.  Radiological and/or clinical criteria | IV.  Clinical deterioration attributed to DCI | V.  Clinical symptoms and vasospasm |
| --- | --- | --- | --- | --- | --- |
| TCD | 6 (23.1%) | 1 (3.8%) | 6 (23.1%) | 7 (26.9%) | 6 (23.1%) |
| NIRS | 0 (0.0%) | 0 (0.0%) | 1 (16.7%) | 2 (33.3%) | 3 (50.0%) |
| EEG | 5 (35.7%) | 0 (0.0%) | 8 (57.1%) | 0 (0.0%) | 1 (7.1%) |
| Total | 11 | 1 | 15 | 9 | 10 |

DCI indicates delayed cerebral ischemia

Across all categories, definitions required exclusion of alternative causes for the observed clinical and/or radiological findings.

### Table S7. Included articles on transcranial doppler

| Articles assessed for eligibility | 75 |  |
| --- | --- | --- |
| Included in review | 26 |  |
| Excluded | 49 |  |
| No sensitivity analysis | | 17 |
| No DCI as reported outcome | | 8 |
| Non English Language | | 2 |
| Not primarily aSAH population | | 1 |
| Published before 2004 | | 20 |
| Vasospasm selected patients | | 1 |

### Table S8. Included articles on near-infrared spectroscopy

| Articles assessed for eligibility | 9 |  |
| --- | --- | --- |
| Included in review | 6 |  |
| Excluded | 3 |  |
| N ≤ 10 | | 3 |

### Table S9. Included articles on electroencephalography

| Articles assessed for eligibility | 27 |  |
| --- | --- | --- |
| Included in review | 14 |  |
| Excluded | 13 |  |
| No sensitivity analysis | | 7 |
| No DCI as reported outcome | | 1 |
| N ≤ 10 | | 1 |
| Non English Language | | 1 |
| Published before 2004 | | 2 |
| no Humans | | 1 |
